# Boosting lithium storage in covalent organic framework via activation of 14-electron redox chemistry

**DOI:** 10.1038/s41467-018-02889-7

**Published:** 2018-02-08

**Authors:** Zhendong Lei, Qinsi Yang, Yi Xu, Siyu Guo, Weiwei Sun, Hao Liu, Li-Ping Lv, Yong Zhang, Yong Wang

**Affiliations:** 1 Department of Chemical Engineering, School of Environmental and Chemical Engineering, Shanghai University, 99 Shangda Road, 200444 Shanghai, China; 2 NUS Graduate School for Integrative Sciences & Engineering, National University of Singapore, Singapore, 117583 Singapore; 3 Institute of Green Chemical Engineering and Clean Energy, Shanghai University, 99 Shangda Road, 200444 Shanghai, China; 4 Department of Biomedical Engineering, National University of Singapore, Singapore, 117583 Singapore

## Abstract

Conjugated polymeric molecules have been heralded as promising electrode materials for the next-generation energy-storage technologies owing to their chemical flexibility at the molecular level, environmental benefit, and cost advantage. However, before any practical implementation takes place, the low capacity, poor structural stability, and sluggish ion/electron diffusion kinetics remain the obstacles that have to be overcome. Here, we report the synthesis of a few-layered two-dimensional covalent organic framework trapped by carbon nanotubes as the anode of lithium-ion batteries. Remarkably, upon activation, this organic electrode delivers a large reversible capacity of 1536 mAh g^−1^ and can sustain 500 cycles at 100 mA g^−1^. Aided by theoretical calculations and electrochemical probing of the electrochemical behavior at different stages of cycling, the storage mechanism is revealed to be governed by 14-electron redox chemistry for a covalent organic framework monomer with one lithium ion per C=N group and six lithium ions per benzene ring. This work may pave the way to the development of high-capacity electrodes for organic rechargeable batteries.

## Abstract (web summary)

Conjugated polymeric molecules are promising electrode materials for batteries. Here the authors show a two-dimensional few-layered covalent organic framework that delivers a large reversible capacity of more than 1500 mAh g^−1^ with the storage mechanism governed by 14-electron redox chemistry.

## Introduction

The application of lithium-ion batteries (LIBs) for energy storage has attracted considerable interest due to their wide use in portable electronics and promising application for high-power electric vehicles^1, 2^. Despite extensive efforts in the synthesis of electrode materials, the rational design of lithium-ion battery electrodes that meet high specific capacity, high-energy density, and outstanding stability remains a challenge^3, 4^. Nowadays, most of the studies focus on the inorganic materials and their carbon-involved composites^5–8^. The organic electrode materials^9–30^ are suggested to be alternative electrode candidates for next-generation LIBs because of their distinct merits compared to the inorganic analogs. The organic electrode materials are potentially low-cost, recyclable, and safe (less exothermic) when fully discharged. More importantly, the understanding of active organic functional groups for efficient lithium storage may accomplish the molecular-level design of the electrode and a large number of new types of electrodes can be developed. Among all types of organic electrode materials, the carbonyl compounds have attracted significant attention as potentially high-capacity cathode or anode materials for lithium-ion batteries. Carbonyl groups can be found in many organic compounds with various forms, while most of the explored carbonyl-based electrodes are mainly based on either quinone or the derivatives of aromatic carboxylic acid^22–24^. Besides, some nonconjugated polymers or layered organic materials have also been explored as promising organic electrodes for LIBs^29, 30^. However, small reversible capacities, the dissolution of electroactive species into organic electrolytes, and poor conductivity of organic electrode materials are serious problems to limit the development of organic electrode materials^31–33^. Covalent organic frameworks (COFs) are a subclass of microporous organic polymers with strong covalent bonds and atomically precise self-assembled two- or three-dimensional morphologies^34–38^. It has been widely used as functional materials for gas adsorption^39^, catalysis^40^, supercapacitors^41–43^, proton conduction^44^, and semiconductors^45^. Among them, the two-dimensional (2D-)layered COFs^46–48^ exhibit the functional *π*-electron systems in the layer for charge transportation and open channels along the direction of layer stacking. Until now, few 2D-layered COF materials have been reported with reversible lithium-storage capacities of ~100–800 mAh g^−1^ when used as the electrode materials for LIBs^25, 49–52^. However, closely packed 2D layer structure of COFs, especially in an eclipsed stacking fashion with strong *π*–*π* interactions, results in the difficulty of lithium-ion infiltration into the interior active sites deeply buried between layers even at a high current density. It will inevitably lead to the insufficient utilization of the redox-active sites and thus lower the lithium-storage capacity of 2D COFs. Mechanical exfoliation of the layered organic structure, which is similar to the exfoliated graphene materials from graphite, has been suggested as an effective strategy to overcome this issue^52, 53^.

Herein, this work shows the controlled growth of few-layered 2D COFs achieved on carbon nanotube (CNT) for application in lithium-ion batteries. In this way, COF is in situ separated by CNT in the growth process and few-layered COF can be obtained around CNT by *π*–*π* interaction between these two components. Lithium-storage redox reactions are associated with not only common C=N groups but also intriguing benzene rings (C=C) of the few-layered COF. The efficient utilization of six carbon atoms of the benzene ring for storage of six lithium ions results in the extremely large capacity contribution of 1536 mAh g^−1^ for COF monomer after repetitive 500 cycles.

## Results

### Structural characterizations

**Fig. 1:**
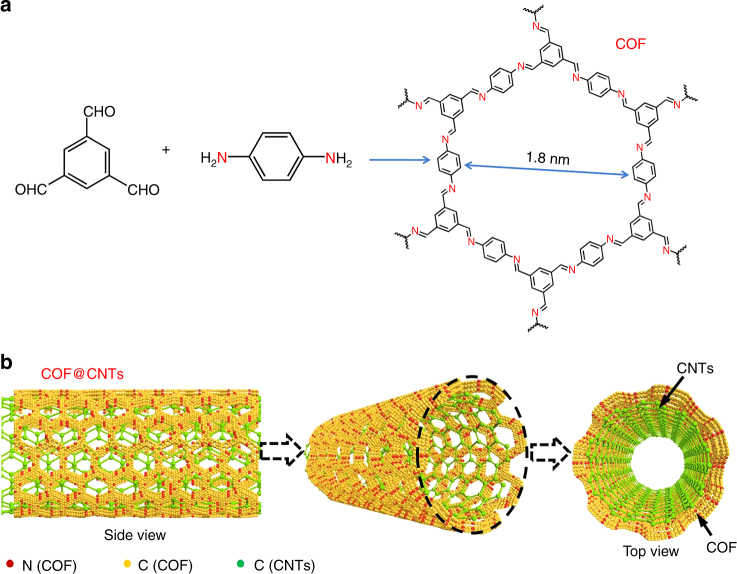
Construction of COF and COF@CNTs. **a** Construction of COF. **b** Graphical representation of COF@CNTs with few COF layers covered on the exterior surface of CNTs

**Fig. 2:**
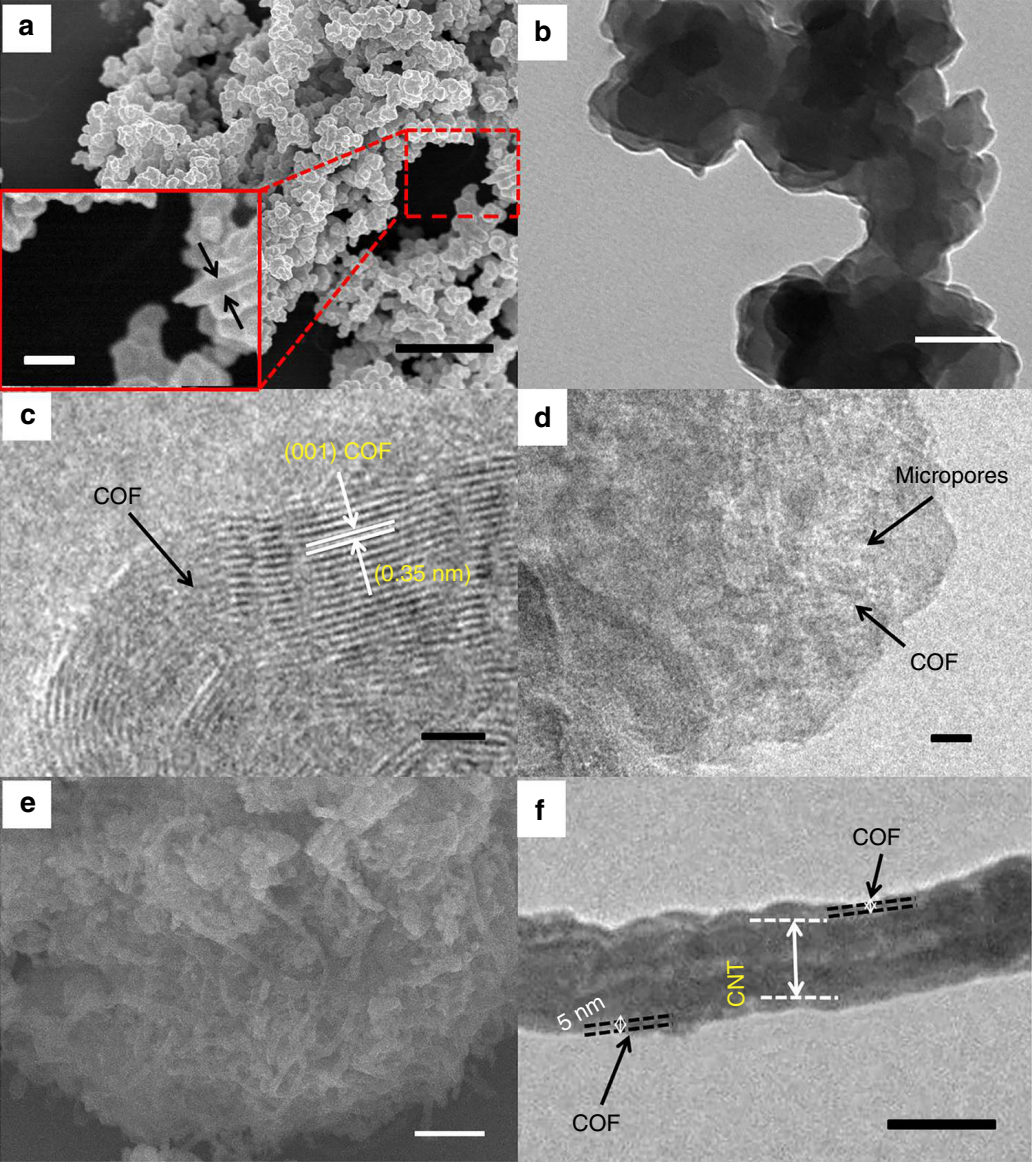
Morphologies of COF and COF@CNTs. **a** SEM images of COF. Scale bar, 1 μm (inset: scale bar, 200 nm). **b** TEM image of COF. Scale bar, 100 nm. **c**, **d** HRTEM images of COF. Scale bar, 5 nm. **e** SEM image of COF@CNTs. Scale bar, 1 μm. **f** TEM image of COF@CNTs. Scale bar, 100 nm

The synthesis of imine-based C=N coupled to COF is illuminated in Fig. 1a. This COF with 2D structure and average pore size of 1.8 nm, was composited with multiwall CNTs to fabricate the COF@CNTs composite with few-layered COF covered on the exterior surface of CNTs (Fig. 1b). The strong C=N stretching peak can be observed at 1618 cm^−1^ in the FTIR spectrum of COF (Supplementary Fig. 1), indicating the formation of imine bonds. Besides, two tiny peaks around 1695 and 3415 cm^−1^ can be assigned to the terminal aldehyde and amino groups of the synthesized COFs. The FTIR spectrum of COF@CNTs is in accordance with that of COF. As shown in Supplementary Fig. 2, the strong diffraction peak at 4.7° in the X-ray diffraction (XRD) patterns can be assigned to the (100) plane of COF, which is in accordance with the simulated results. Based on the Bragg equation, the *d* value along the *a* direction can be calculated to be ~1.8 nm, which agrees well with the calculated pore size of COFs. A tiny peak at 25.5° can also be detected for COF, which can be assigned to the (001) plane of COF. Besides, several small peaks at ~8.1°, 9.4°, and 12.7° in the simulated XRD patterns for COF cannot be observed in the XRD results for as-synthesized COF^54, 55^. It might be attributed to the lack of long-distance order in the as-synthesized COF prepared after a short reaction time. A strong peak around 26.1° can be observed for COF@CNTs, which should be assigned to the (002) plane of CNTs. The peak (at 25.5°) assigned to the (001) plane of COF cannot be observed for the COF@CNTs due to the overlap of the strong peak (~26.1°) for CNTs. The thermal stability of the COF and COF@CNTs is shown in TGA curves of Supplementary Fig. 3. COF and COF@CNTs exhibit good thermal stability up to 400 °C in air. From the nitrogen adsorption and desorption measurement, a Brunauer–Emmett–Teller (BET) surface area of 44.36 m^2^ g^−1^ and the pore size distribution at ~1.4–1.6 nm are detected for COF, while a larger BET surface area (52.73 m^2^ g^−1^) and the similar pore size distribution centered at ~1.5 nm are determined for the COF@CNTs composite (Supplementary Fig. 4). It is worth noting that the pore size distribution of COF detected from BET results is slightly smaller than the value (1.8 nm) calculated based on the XRD result, which is possibly due to the partly occupied pores induced by staggered stacking of the 2D COF layers along the *c* direction. The adoption of the COF with a small surface area is aimed to obtain short-range ordered layered structure with more defects, which can facilitate lithium diffusion. For comparison, the products of COF-3 days and COF@CNTs-3 days with improved crystallinity and larger surface areas were also obtained via similar synthesis procedures with prolonged synthetic time of 3 days (Supplementary Fig. 5) The scanning electron microscopy (SEM), transmission electron microscopy (TEM), and high-resolution TEM (HRTEM) reveal the stacked 2D lamellar structure with micropores along the lamellar stacking direction for the as-synthesized COF (Fig. 2a–d). The interplanar distance of ~0.35 nm can be clearly observed from the HRTEM image of COF, which is in accordance with the *d* value calculated based on the peak (25.5°) in XRD pattern for COF^55^. In the presence of CNTs, the COF@CNTs composite delivers a thin COF layer (~5 nm in thickness) covered on the exterior surface of CNTs (average diameter in the range of 50–80 nm) (Fig. 2e, f).

### Electrochemical properties characterizations

**Fig. 3:**
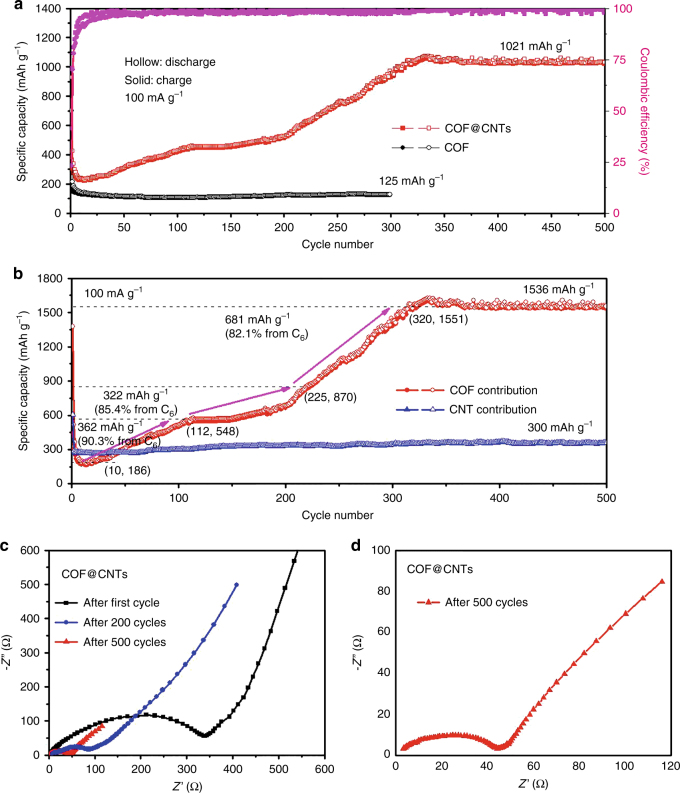
Electrochemical performances of the COF@CNTs anode. **a** Cycling performances of the COF and COF@CNTs at 100 mA g^−1^. **b** Capacity contribution of COF (based on the mass of COF) in COF@CNTs at 100 mA g^−1^. **c** Nyquist plots of the COF@CNTs after the first 200 and 500 cycles. **d** The enlarged Nyquist plots after the 500th cycle

As indicated from the first-cycle cyclic voltammetry (CV) curve of COF@CNTs (Supplementary Fig. 6a), a detected peak at ~1.5 V in the first cathodic scan can be attributed to the lithiation reaction with the C=N functional group. Several small reduction peaks observed at ~0.72, 0.12, and 0.06 V can be ascribed to several lithiation reactions with the C=C groups of benzene rings, as well as the formation of a solid electrolyte interphase (SEI) film^13^. The corresponding anodic peaks at ~0.75 and ~1.6 V for the first cycle can be identified in the delithiation process^25, 50^. The first discharge–charge profile of the COF@CNTs is shown in Supplementary Fig. 6b, which is in good accordance with the CV results. The initial discharge (lithiation) and charge (delithiation) capacities of the COF@CNTs anode are detected to be 928 and 383 mAh g^−1^. The calculated low Coulombic efficiency of 41.3% in the first cycle is mainly ascribed to the electrolyte decomposition and the formation of the SEI film. The discharge/charge capacities of the COF@CNTs anode (Fig. 3a) are increased to 768/763 and 1032/1021 mAh g^−1^ after 260 and 500 cycles, respectively. The pristine bulk COF exhibits stable cycling performance with a small reversible capacity of ~125 mAh g^−1^ achieved at the 300th cycle. The discharge and charge curves of the bulk COF anode for various cycles are provided for comparison in Supplementary Fig. 6c. In comparison, the COF@CNTs composite anode exhibits larger reversible capacities during 500 cycles. After the capacity decreases to ~230 mAh g^−1^ for the initial ten cycles, the reversible capacities increase to 443 mAh g^−1^ from the 10th to 112th cycle and keep a moderate increase up to the 225th cycle. After the subsequent significant capacity increase from the 225th cycle to the 320th cycle, an extremely large reversible capacity of 1021 mAh g^−1^ is achieved for the COF@CNTs composite and there is almost no capacity fading during the following cycles. The composite capacity of COF@CNTs can be regarded as the contribution from two components of COF and CNTs, and the CNTs exhibit a reversible capacity of ~300 mAh g^−1^ without substantial capacity fading during long-term cycling (Fig. 3b). Therefore, the capacity contribution from COF in the composite can be calculated based on the weight ratio of COF and CNTs (1.4:1). The composition was estimated based on the N and C contents in the pristine COF, CNTs, and COF@CNTs. The elemental analysis results and more details relative to the composition calculation are provided in Supplementary Table 1 and Supplementary Note 1. A large reversible capacity contribution of 1536 mAh g^−1^ is achieved for the COF in the composite, which is even higher than the theoretical capacities (700–1000 mAh g^−1^) of inorganic anodes such as transitional metal oxides (CuO, Fe_2_O_3_, Co_3_O_4_, NiO, etc.) and Sn-based anodes. The rate performances were also conducted on the fully activated COF@CNTs electrode (Supplementary Fig. 7). As determined by the electrochemical impedance spectroscopy (EIS) results (Fig. 3c, d), the charge-transfer resistance (*R*_ct_) of COF@CNTs anode (337 Ω for the first cycle) decreases to be 87 Ω after 200 cycles, and 44 Ω after 500 cycles, confirming the gradual activation process during cycling. As shown in Supplementary Fig. 8, inferior lithium-storage properties can be detected for the products of COF-3 days and COF@CNTs-3 days. A similar phenomenon (better Li-storage properties in COF with more amorphous structure) was also observed in the previous report^52^.

### Lithium-storage mechanism

**Fig. 4:**
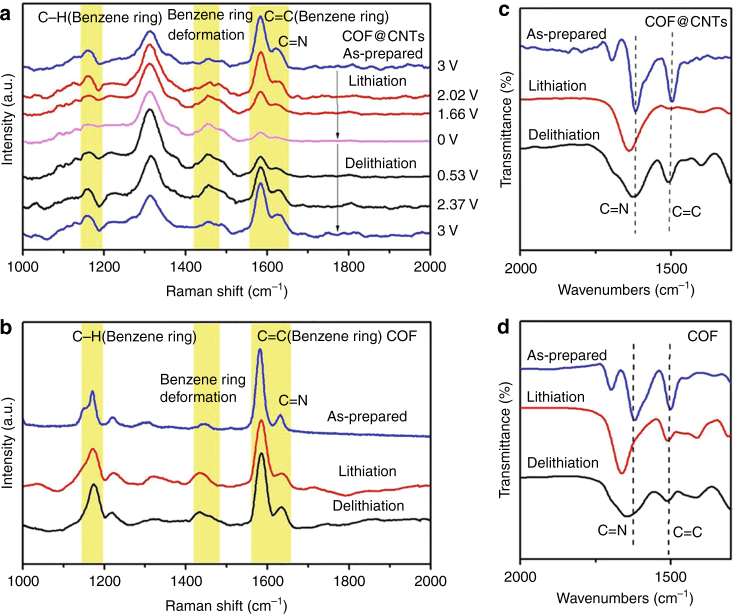
Structural evolution characterization of COF@CNTs and COF anodes. **a** Raman spectra of as-prepared COF@CNTs and COF@CNTs anode during lithiation (discharge) and delithiation (charge) process. **b** Raman spectra of as-prepared COF and COF anode during lithiation and delithiation process. **c** FTIR characterizations of the as-prepared COF@CNTs and COF@CNTs anode during lithiation and delithiation process. **d** FTIR characterizations of the as-prepared COF and COF anode during lithiation and delithiation process

**Fig. 5:**
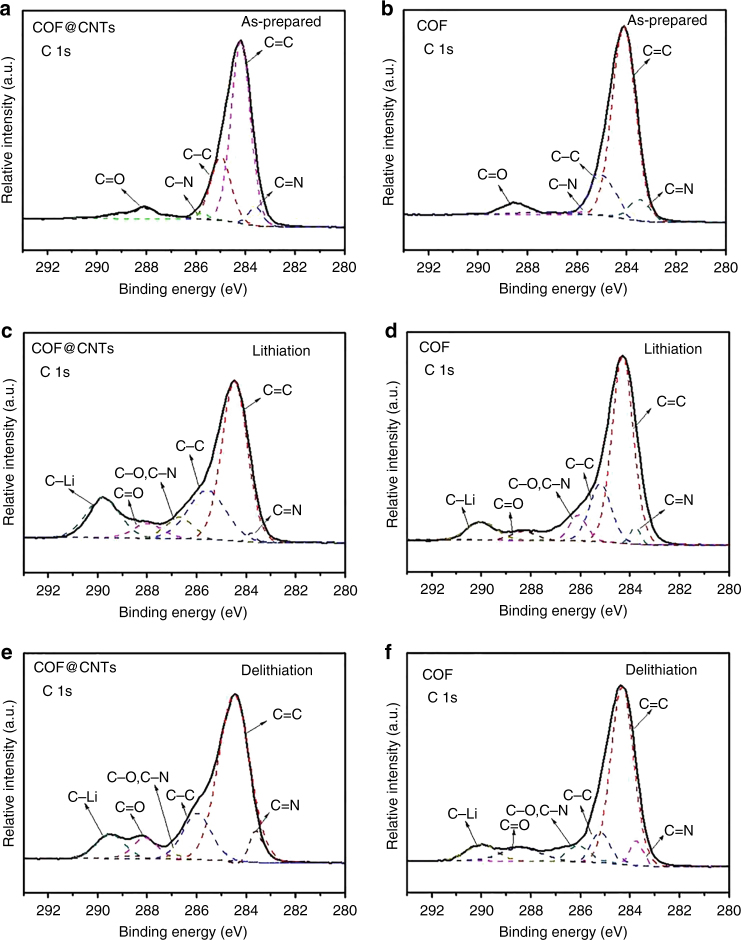
XPS results for COF@CNTs and COF. **a** C 1s scan of as-prepared COF@CNTs, **b** C 1s scan of as-prepared COF, **c** C 1s scan of COF@CNTs after lithiation process, **d** C 1s scan of COF after lithiation process, **e** C 1s scan of COF@CNTs after delithiation process, and **f** C 1s scan of COF after delithiation process

A larger capacity contribution (1536 mAh g^−1^) is observed for COF in the COF@CNTs composite compared to bulk COF (125 mAh g^−1^). It is indicated that the functional groups of the COFs exhibit different electrochemical activities in two cases. Therefore, a series of characterization techniques are used to gain insight into the lithium-storage mechanism. Raman/FTIR measurements are conducted on the as-prepared COF and COF@CNTs, as well as on two anode materials during the first fully discharged (lithiation) process to almost 0 V (5 mV) and the first fully charged (delithiation) process to 3 V. As illuminated in the in situ Raman results for COF@CNTs and pristine bulk COF anode (Fig. 4a, b), several bands at ~1630, ~1580, ~1450, and ~1160 cm^−1^ can be assigned to the C=N groups, the aromatic C=C groups from benzene rings, the deformation vibration of benzene rings, and the C–H groups from benzene rings, respectively. The band at ~1320 cm^−1^ can be ascribed to the D band of CNTs. It is observed that the band of C=N groups for COF@CNTs anode becomes gradually weaker and then almost disappears in the lithiation process, while it gradually recovers during the following delithiation process. It should be assigned to the reversible lithium-ion reactions with the C=N groups of COF monomer in the COF@CNTs anode (Fig. 4a). In comparison, the C=N band slightly weakens after the lithiation process and then recovers after the delithiation process for pristine COF anode, indicating partial participation of its C=N groups for lithium storage (Fig. 4b). Besides, the redshift can be observed for the peak of C=N stretching modes (from 1618 to 1638 cm^−1^) after the lithiation process and its recovery can be detected after delithiation, as shown in ex situ FTIR spectra of the as-prepared and the cycled COF@CNTs anode (Fig. 4c). Similar variation can also be detected for pristine COF anode. These observations confirm the lithium storage with C=N functional groups, which has also been reported for previous organic electrodes with the lithium-storage mechanism of one lithium per C=N group^18–21^. Therefore, more attention has been paid to the lithium-storage properties on the C_6_ benzene rings with the C=C functional groups. As shown in Fig. 4a, the band (assigned to C=C groups) around 1570 cm^−1^ becomes obviously weak during the lithiation process of COF@CNTs anode and then almost disappears when discharged to 0 V. During the following delithiation process, this band appears and becomes stronger after recharging to 3 V. It indicates the reversible C=C bonds destruction with Li-ion insertion and its restoration with Li-ion extraction. The same variation trend can also be observed for the FTIR peak of aromatic C=C stretching modes in benzene rings (at ~1506 cm^−1^), as shown in the ex situ FTIR spectra of Fig. 4c. It is indicated that the C=C groups of benzene rings in the COF@CNTs anode participate in the redox reactions with lithium ions, which has not been witnessed in previous COF-based electrodes^25, 49–52^. In comparison, the change of C=C groups of benzene rings during cycling cannot be detected for pristine COF anode, based on the Raman and FTIR results (Fig. 4b, d). It should be ascribed to the fact that the few-layered COF in the COF@CNTs composite is in favor of more lithium-ion insertion into the interlamination of the 2D lamellar COF structure, which may facilitate the lithium reaction with more functional units compared to the pristine COF material^52^. Along with the change of C=C groups from benzene rings, the corresponding C–H group variation and benzene ring deformation can also be detected during the lithiation/delithiation process of the COF@CNTs anode, as indicated from the Raman spectra (Fig. 4a). However, these changes of two bands are indiscernible for bulk COF anode, further confirming the Li-storage mechanism on C=C of benzene rings for the few-layered COF in the composite rather than pristine COF. X-ray photoelectron spectroscopy (XPS) was conducted on the COF@CNTs and pristine bulk COF to probe their Li-storage mechanism (Fig. 5). As shown in Fig. 5a, b, the C 1s spectra for both as-prepared COF@CNTs and pristine COF can be deconvoluted to five peaks centered at around 288.2, 285.8, 284.9, 284.2, and 283.5 eV, which correspond to C=O, C–N, C–C, C=C, and C=N groups, respectively^28^. After the lithiation (discharge) process (Fig. 5c), a new peak (289.6 eV) indexed to C–Li groups appears^51^, which results from the lithiation reaction with the aromatic C=C groups in C_6_ benzene rings in the COF@CNTs anode and the formation of an SEI layer. This peak is obviously weakened in the following charge process (Fig. 5e), indicating the reversible delithiation reaction on the aromatic C=C groups. This phenomenon cannot be detected for pristine COF (Fig. 5d, f), confirming the inactive aromatic C=C groups in pristine COF anode. Furthermore, the area ratio of the peak for C–C and C=C groups increases during the lithiation process and then recovers in the following delithiation process (Fig. 5c, e), indicating the reversible transformation between C=C and C–C groups in benzene rings from COF structure. This phenomenon cannot be observed for pristine COF anode (Fig. 5d, f), further confirming the absence of benzene rings of pristine COF anode in the lithium storage. It is worth noting that the tiny peak at ~288.2 eV detected for the COF@CNTs and COF should be assigned to the small amount of residual C=O groups from terminal aldehyde units existed in the synthesized COF, and it corresponds to the lithium-reversible reaction with C=O groups (transformation between C=O and C–O groups). It is in good accordance with the detected tiny C=O peak at 1695 cm^−1^ from the FTIR results of the as-prepared and the cycled COF@CNTs and COF anodes (Fig. 4c, d). This tiny C=O peak disappears during lithiation process and recovers as a shoulder peak of C=N peak after full delithiation. Because there is only a trace amount of the terminal C=O groups, the Li-storage associated with these groups is not considered during the calculation on the capacity contribution of COF@CNTs.

**Fig. 6:**
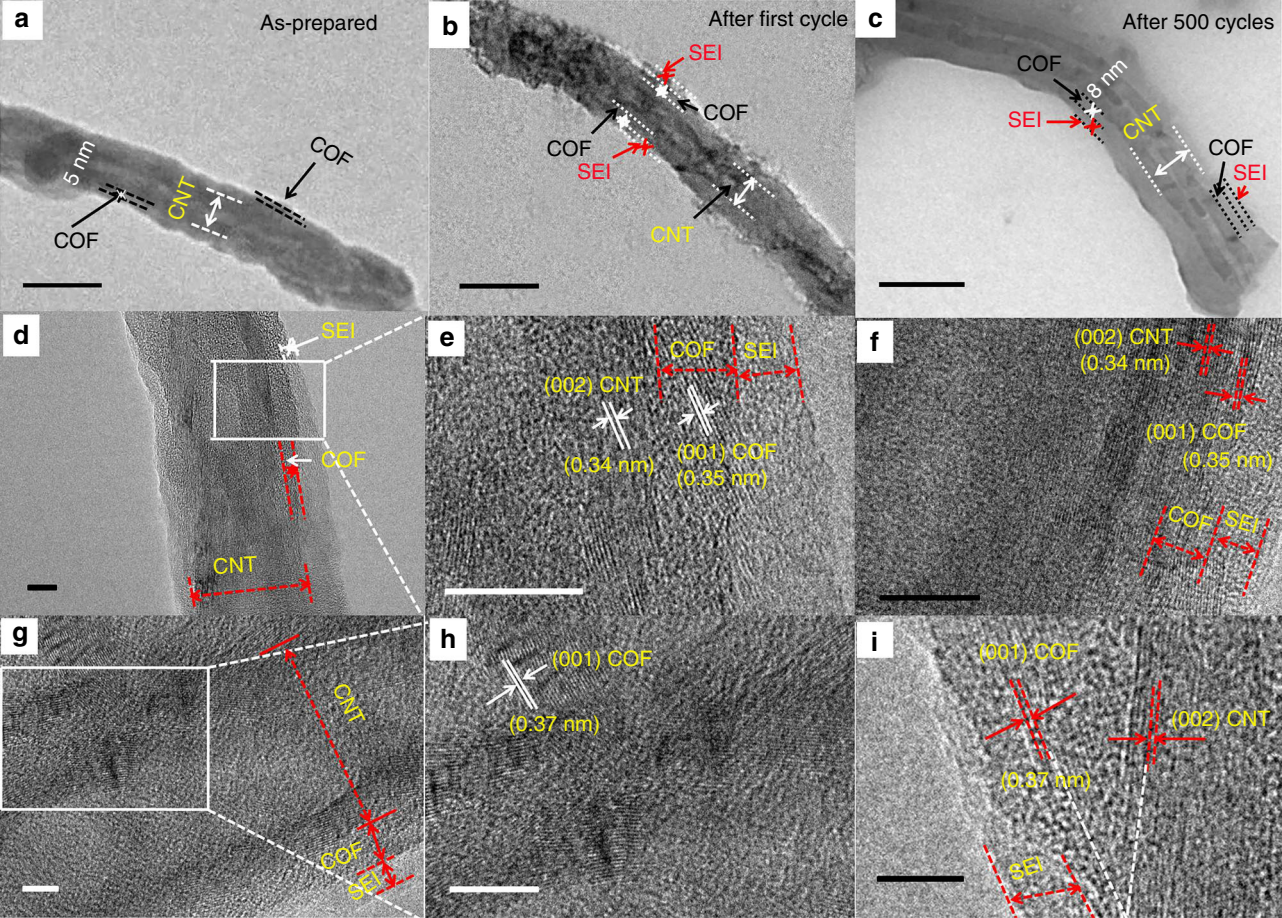
Morphology variations of COF@CNTs anode during cycling. TEM images of COF@CNTs: **a** as-prepared, **b** after the first cycle, and **c** after 500 cycles. Scale bar, 100 nm. **d**–**f** HRTEM images of COF@CNTs after the first cycle. Scale bar, 10 nm. **g**–**i** HRTEM images of COF@CNTs after 500 cycles. Scale bar, 5 nm

**Fig. 7:**
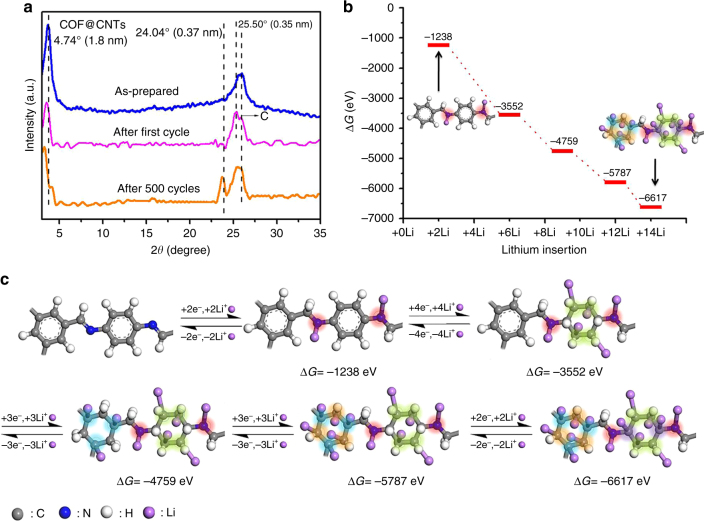
Structural variations and the density functional theory (DFT) calculation of COF in COF@CNTs anode. **a** XRD patterns of as-prepared COF@CNTs and the COF@CNTs anode after the first cycle and 500 cycles. **b** Reaction free energies (Δ*G*) at various stages of the lithiated COF monomer. Reaction free energies were determined by subtracting the minimized energies of the lithiated COF monomer at each lithiation stage from those of the initial compounds and lithium. **c** The structure evolution during the lithiation process. The binding sites between lithium and COF are highlighted with red, yellow, blue, orange, and purple colors, respectively

Furthermore, TEM images are used to provide insights into the structural evolution in the COF@CNTs anode during cycling (as-prepared, after the first cycle and after 500 cycles, respectively, in Fig. 6a–c). A thin layer of SEI appears around COF layer after the first cycle and the COF layer becomes very thick after 500 cycles. More microstructural details can be determined from HRTEM images of Fig. 6d–i. Compared with the as-prepared COF@CNTs, the structure of COF layer (~5 nm in thickness) trapped on the exterior surface of CNTs can be maintained with a thin SEI layer coverage (~3–5 nm in thickness) after the first discharge/charge cycle (Fig. 6d). The interplanar distances of ~0.35 nm and ~0.34 nm can be assigned to the (001) lattice plane of COF and (002) lattice plane of CNTs, respectively (Fig. 6e, f). It is worth noting that the parallel lattice direction can be observed for CNTs and the covered COF layer, indicating that the 2D lamellar structure of COF wrapped perfectly to the exterior walls of CNTs under the interaction of extended *π* bonds from COF and CNTs. The molecular dynamics (MD) simulation was conducted on the COF@CNTs composite via the Materials Studio (MS) software to confirm the COF coverage over CNTs with the *π* interaction between them, as displayed in Supplementary Fig. 9. HRTEM images of COF@CNTs anode after 500 cycles are shown in Fig. 6g, h, which reveal the thickened COF layer (~8 nm) with increased interplanar distance of ~0.37 nm. As shown in Fig. 6i, a thicker COF layer can be detected, which should be ascribed to the partial expansion of COF layer on the exterior surface of CNTs. The interlamellar spacing expansion of COF lamellar structure should be ascribed to repetitive lithium insertion and extraction. The determination details of *d* value are provided in Supplementary Fig. 10, in which the magnified lattice spacing of ten lattice fringes was measured to calculate the accurate *d* value for COF. The expanded change of *d* value along the *c* direction can also be confirmed based on the calculation from the XRD characteristic (001) peak for the COF@CNTs anode after cycling. As shown in Fig. 7a, the XRD peak of 25.50° assigned to the (001) planes of COF can be observed for the COF@CNTs anode after the first discharge/charge cycle. After 500 cycles, this XRD peak is shifted to 24.04° with the calculated *d* value of ~0.37 nm. Moreover, the XRD peak for (100) plane of COF (~4.74°) is also slightly shifted to a smaller angle, indicating the slight increase in pore size of COF during repetitive lithiation/delithiation process.

**Fig. 8:**
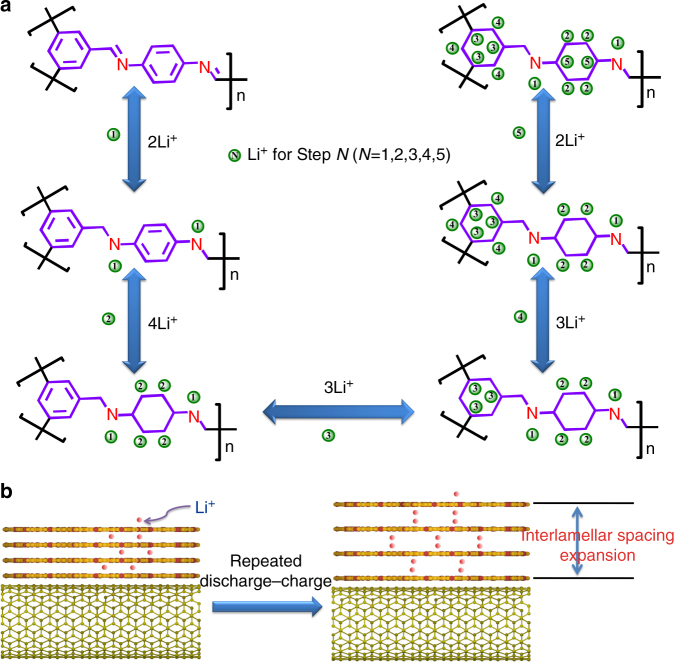
Schematical illustration showing stepwise lithium-storage mechanism for COF in the COF@CNTs anode. **a** Reversible five-step lithium-ion insertion and extraction reactions with a COF monomer. **b** The facilitated lithium-ion transport and storage into expanded COF layers during the repeated lithiation and delithiation process

The lithium-storage mechanism is investigated theoretically by the density functional theory (DFT) calculation (Fig. 7b, c and Supplementary Fig. 11). In per-COF monomer, two possible adsorption sites for lithium are considered: 12 C atoms from two benzene rings and two N atoms from two C=N functional groups. Based on the hypostasized 14-lithium-ion storage for per-COF monomer, the binding energy of per Li^+^ is calculated to be 5.16 eV when two lithium ions are stored with two C=N groups, while it decreases to 1.75 eV when all the 14 sites of a COF monomer are occupied by lithium ions. For the conjugated system between the benzene ring and its neighboring C=N group, the electron-withdrawing N atom is in favor of its connection with metal ions, resulting in the preferred lithium insertion in C=N group rather than the benzene ring. Besides, both the reaction free energies for lithium addition on the C=N groups and benzene rings of COF monomer were calculated based on DFT simulation (Fig. 7b), indicating that the stepwise lithiation reaction with C=N groups and benzene rings of the COF@CNTs anode is reasonable. Significantly lower free energy is achieved for 14-Li-inserted COF anode than for unsubstituted COF. Furthermore, the stepwise lithiation reaction with benzene rings of COF monomer has been probed based on the C atoms from benzene rings with different structural conditions. After the initial insertion of two Li ions with two C=N groups, it is preferential that four lithium ions are bound with four unsubstituted carbons of the two-substituted benzene ring highlighted in yellow (Fig. 7c) due to the lowest total energy under this condition. Then, the lithium-ion insertion on benzene rings follows the following order based on the comparison of their total energy calculations: three lithium ions on the three-substituted carbons of the three-substituted benzene ring highlighted in blue, three lithium ions on the other three unsubstituted carbons of the three-substituted benzene ring highlighted in orange, and finally, two lithium ions on the two-substituted carbons of the two-substituted benzene ring highlighted in purple. The morphological change of the COF wrapping over CNTs in the COF@CNTs during the five-step lithium-storage process has also been illuminated by the MD simulation (Supplementary Fig. 12). This fourteen-lithium-storage mechanism based on the five-step lithiation/delithiation process for a COF monomer in COF@CNTs is illuminated in Fig. 8a. The achievement of fourteen lithium-storage mechanism for the COF@CNTs anode after long cycling should be ascribed to its interlamellar spacing expansion, as indicated in Fig. 8b. With the expanded spacing, more lithium ions can get access into the interlamellar space of the COF layer during cycling, leading to the gradually facilitated lithiation/delithiation kinetics at benzene rings. There is a clear change with C=N groups but no detected change for aromatic C=C groups for pristine bulk COF based on the Raman, FTIR, and XPS results. It is believed that the lithiation/delithiation at benzene rings indicated by steps 2–5 is inactive for pristine bulk COF. Based on the above results and observation, the astonishingly high Li-storage capacity contribution of COF component may be explained by the 14-lithium-ion storage per monomer unit of COF. Besides two lithium ions stored based on the reversible redox reactions on two C=N groups, a maximum of 12 lithium ions can be stored on the unsaturated carbons of two benzene rings in a COF monomer by forming Li_6_/C_6_. The theoretical capacity of the COF is calculated to be 1830 mAh g^−1^ based on the 14-lithium-storage mechanism. The detected capacity contribution of 1536 mAh g^−1^ for the COF after 500 cycles in this work is around 83.9% of the theoretical value. In comparison, the pristine bulk COF can only deliver a smaller reversible capacity of 125 mAh g^−1^ after 300 cycles, which is around 47.9% of its theoretical capacity of 261 mAh g^−1^ based on two-lithium-storage mechanism. The CV tests at different sweep rates of 1–4 mV s^−1^ were conducted on the as-prepared COF and the COF@CNTs electrode before and after cycling (Supplementary Fig. 13). The linear relation between peak current and the square root of the scan rate can be observed for the as-prepared COF and COF@CNTs electrodes, indicating their diffusion-dominated redox-reaction kinetics (Supplementary Figs 13a and b). The detected linear relation of peak current versus scan rate for the COF@CNTs electrode after 186, 260, and 320 cycles (Supplementary Figs 13c–e) verifies the surface-controlled redox reaction for the COF@CNTs electrode after activation.

**Fig. 9:**
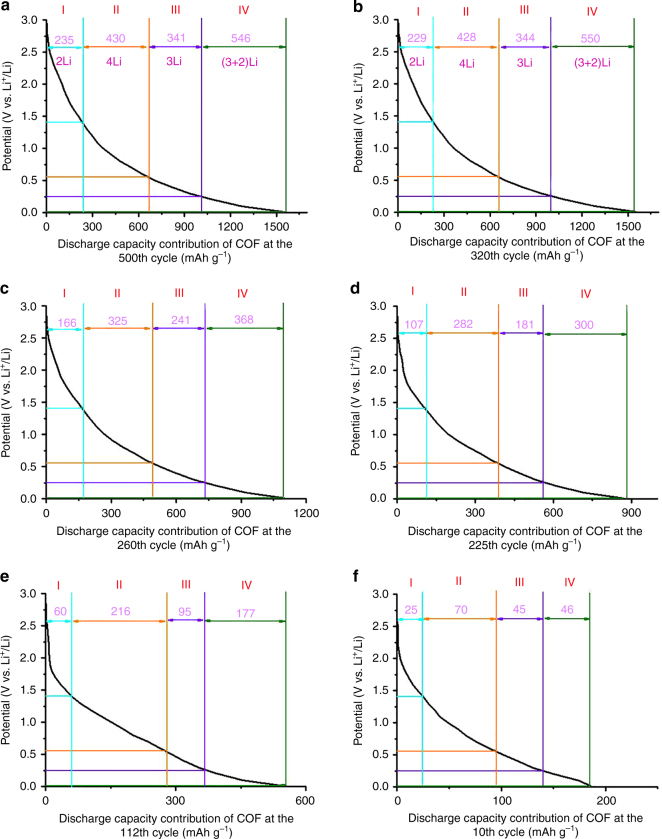
Discharge curves of COF contribution in COF@CNTs anode. The discharge curve for COF contribution at the **a** 500th, **b** 320th, **c** 260th, **d** 225th, **e** 112th, and **f** 10th cycles with stepwise lithium-storage and the corresponding capacity contribution is marked

In order to investigate the gradual activation process, further exploration was performed about the stepwise lithium-storage contribution from C=N groups and benzene rings. As indicated by Fig. 3b, there are three major capacity increase periods (a detected capacity increase from the 10th to the 112th cycle, followed by a moderate increase between the 112th and the 225th cycle and a significant increase from the 225th to the 320th cycle) and the capacity is quite stable during 320–500 cycles. Supported by the discharge curves for COF@CNTs composite and pristine CNTs as shown in Supplementary Table 2, the discharge capacity contribution of COF in COF@CNTs at different potentials during these critical cycles can be calculated. The discharge curves of COF contribution at the 10th, 112th, 225th, 260th, 320th, and 500th cycles in the voltage range of 5 mV–3 V are selected and illuminated in Fig. 9 with the capacity data listed in Supplementary Table 3. Due to the ultrahigh discharge capacity of 1552 mAh g^−1^ detected for the COF contribution at the 500th cycle, the 14-lithium-ion storage is roughly divided into five stages based on these experimental data. The experimental discharge capacity for per-Li ion can be calculated to be 111 mAh g^−1^. A first-stage lithium-ion storage of ~235 mAh g^−1^ can be observed within the potential window of 2.7–1.4 V (Fig. 9a, b), which agrees with two lithium-ion storage on two C=N groups (step I). This voltage potential is also in good accordance with the CV curve in Supplementary Fig. 7a. Therefore, the following steps (II–IV) may be roughly ascribed to the stepwise lithium storage on benzene rings with four Li^+^ storage (step II), three Li^+^ storage (step III), and five Li^+^ storage (step IV) occurred under the potentials of 1.4–0.57, 0.57–0.25, and 0.25–0.005 V, respectively. It is in accordance with the stepwise fourteen-lithium-storage mechanism (Fig. 8a), except for step IV corresponding to three Li^+^ on benzene rings (step 4) and two Li^+^ on benzene ring (step 5) processes as shown in Fig. 8a due to the indistinguishable voltage ranges for these two steps. The galvanostatic intermittent titration technique (GITT) and potentiostatic intermittent titration technique (PITT) measurements have also been conducted on the activated COF@CNTs composite to distinguish its stepwise Li-storage behavior, as shown in Supplementary Fig. 14. Several steps of Li-storage with corresponding two Li^+^ on C=N groups, four Li^+^, and three Li^+^ on benzene rings can be roughly indicated although the last step of the five-Li-ion storage on benzene rings is hard to determine. As shown in Fig. 9a, a capacity of 235 mAh g^−1^ for lithium-storage on C=N groups, as well as the capacity of 1317 mAh g^−1^ for lithium-storage on benzene rings can be detected at the 500th cycle. The capacity contribution percentage of lithiation by benzene rings is ~84.9%. Compared to the data of the 500th cycle, there is almost no change of capacity contribution during different voltage windows for the 320th cycle (Fig. 9b). To get insight into the capacity contribution of lithium-storage on C=N groups and benzene rings during cycling, the discharge curves of COF contribution at the critical cycles are shown in Fig. 9b–f. There are significant capacity increases during different stages, especiallly for the low-potential windows. The proportions of lithium-storage capacity contribution from benzene rings are determined to be ~85%, ~88%, ~89%, and ~87% at the 260th, 225th, 112th, and 10th cycles, respectively. The total capacity increments for the COF during the capacity increase stages with the capacity increment contribution from both C=N groups and benzene rings are listed in Supplementary Table 4, which indicates that the major contribution of capacity increments also arises from lithium-storage on benzene rings. The capacity contribution of lithium-storage on C=N groups from COF can be detected to be 166, 107, 60, and 25 mAh g^–1^ at the 260th, 225th, 112th, and 10th cycles, respectively, which correspond to the ~75%, ~49%, ~27%, and ~11% of the capacity for the full two-lithium-ion storage on C=N groups, respectively. It is indicated that both C=N groups and benzene rings undergo a gradual activation process during repetitive cycling.

There are only several COF-relative materials, which have been suggested as potential electrodes for LIBs due to their micro/mesoporous structure and active functional groups with lithium ions, such as carbonyl and imine groups.^25, 49–52^ As listed in Supplementary Table 5, the COF materials containing carbonyl groups are usually explored as the cathodic materials based on the lithium reaction with C=O units^25, 49, 52^. This Li-storage mechanism is also common for most organic electrodes^29^. Besides, the existence of imine units and/or aromatic heterocyclic groups promotes the usage of COF materials as the anodes for LIBs^50, 51^. Furthermore, few-layered 2D COF structures, which have been obtained by mechanical exfoliation of ball milling, are reported with obviously improved electrochemical performance with more lithium ion insertion^52^. It is worth noting that the benzene rings that involved Li-storage mechanism have not been demonstrated for previous COF-relative electrode materials. Compared to one-lithium-ion storage with per C=O or C=N group, the one-lithium-ion storage with one carbon of the benzene rings can offer substantially larger theoretical specific capacity due to smaller molecular weight for one carbon atom as compared to that in Supplementary Table 5.

## Discussion

In conclusion, a synthetic COF@CNTs composite is synthesized via a facile room-temperature method and used as the anode material for lithium-ion batteries. The COF@CNTs exhibit excellent electrochemical performance with gradually increased reversible capacity during cycling before ~320 cycles and a large and stable reversible capacity of 1021 mAh g^−1^ can be achieved during ~320–500 cycles. This capacity value can be referred to the capacity contribution of 1536 mAh g^−1^ from the COF in COF@CNTs composite. Based on the electrode characterizations, the DFT calculation, and electrochemical analysis at different voltages during various cycles, the lithium-ion storage mechanism of COF@CNTs is suggested to be 14-lithium-storage for a COF monomer with one lithium ion on per C=N group and six lithium ions on per benzene ring. The few-layer lamellar-conjugated COF structure on electrically conductive CNT along with gradual interlamellar spacing expansion induced by repetitive lithium insertion is beneficial for the efficient achievement of 14-lithium-ion storage of COF monomer. Further work is certainly needed to improve the slow kinetics of lithium insertion into COF.

## Methods

### Materials

1,3,5-Benzenetricarboxaldehyde (Sigma-Aldrich, 95%), 1,4-diaminobenzene (AR, 97%), carbon nanotubes (MWCNTs, *Φ* = 50–80 nm), 1,4-dioxane (AR, 99%), *N*,*N*-dimethylformamide (AR, 99.5%), and tetrahydrofuran (AR, 99%).

### Synthesis of COF and COF@CNTs

1,3,5-Benzenetricarboxaldehyde (16 mg), CNTs (16 mg), and 1,4-diaminobenzene (16 mg) were dispersed in 1.0 mL of 1,4-dioxane. A volume of 0.2 mL of 3.0 mol/L aqueous acetic acid was then added into the suspension. After further stirring for 5 h at room temperature (25 °C), green solid precipitates were obtained. After centrifugation, copious washing with *N*,*N*-dimethylformamide and tetrahydrofuran, Soxhlet extraction (THF as solvent), and drying at 60 °C under vacuum for 12 h, the final product of green-colored COF@CNTs was collected. The pristine bulk COF was also synthesized in the similar process but in the absence of CNTs. The products of COF-3 days and COF@CNTs-3 days were obtained through a similar procedure with prolonged reaction time of 3 days.

### Fourier transform-infrared spectrometer measurements

Fourier transform-infrared spectra were collected on a FTIR spectrophotometer (BIO-RAD FTS 135).

### X-ray diffraction

Powder XRD patterns were collected on Rigaku D/max-2550V (CuK_α_ radiation).

### Thermal gravimetric analyzer measurements

Thermogravimetric analysis was characterized in air at a ramp rate of 10 °C min^−1^ on a NETZSCH STA 409 PG/PC instrument.

### BET measurements

The BET surface area and pore size distributions were measured on an accelerated surface area and porosimetry analyzer (Micromeritics, ASAP 2020M+C, N_2_).

### X-ray photoelectron spectroscopy

The elemental valences of the products were characterized by XPS (PHI ESCA-5000C).

### Field-emission scanning electron microscopy

Field-emission scanning electron microscopy (FE-SEM) images were acquired by a field-emission scanning electron microscopy (FE-SEM, JSM-6700F).

### Transmission electron microscopy

TEM images were acquired by two TEM instruments (JEOL JEM-200CX and JEM-2010F).

### In situ Raman analysis

Raman spectroscopy was collected on Renishaw plus laser Raman spectrometer (wavelength: 785 nm and power: 3 mW). The active-electrode material was coated on the copper mesh and loaded into an in situ analytical lithium battery test device (STC-ZINGAIR-W). The Raman’s laser can transmit through the quartz glass window of the test device, which is connected with a LAND-CT2001C test system for electrochemical measurements.

### Elemental analysis

CHN elemental analysis was measured by Elementar Vario MICRO cube.

### Electrochemical measurements

The Swagelok-type cells were assembled in an argon-filled glove box for electrochemical measurements. Lithium foil was used as the reference and counter electrode. The mixture of 80 wt% of the COF@CNTs composite, 10 wt% of acetylene black, and 10 wt% of polyvinylidene difluoride (PVDF) (binder) was used as the working electrode. The electrode material of the working electrode was loaded onto the current collector of copper foil with a loading amount of 2 mg cm^−2^ and the electrode thickness of ~20 μm. The specific capacity was based on the total mass of the composite electrode materials. Around 0.15 mL of 1 M LiPF_6_ dissolved in ethylene carbonate and diethyl carbonate (1:1 w/w) was used as the electrolyte. The cells were lithiated and delithiated at different currents in the fixed potential range (5 mV–3.0 V vs. Li^+^/Li) on a LAND-CT2001C system. An electrochemical workstation (CHI660D) was used to perform cyclic voltammetry (CV) (0.1 mV s^−1^) and Nyquist plots (100 kHz–10 mHz). GITT and PITT measurements were conducted using the AUTOLAB PGSTAT101 electrochemical workstation and the LAND-CT2001C system.

### Capacity contribution of COF in COF@CNTs calculation

The composite capacity of COF@CNTs can be regarded as the contribution of two components, as described by the following equation:

1 $${\it{C}}_{{\mathrm{COF@CNTs}}} = {\it{C}}_{{\mathrm{COF}}} \times {\it{P}}_{{\mathrm{COF}}} + {\it{C}}_{{\mathrm{CNTs}}} \times {\it{P}}_{{\mathrm{CNTs}}}$$

where *C*_COF@CNTs_, *C*_COF_, and *C*_CNTs_ represent the capacities of COF@CNTs, COF, and CNTs, respectively. *P*_COF_ and *P*_CNTs_ correspond to the mass percent of COF and CNTs in the COF@CNTs composite.

### DFT simulation

The theoretical calculations were based on the DFT in conjunction with the projector-augmented-wave (PAW) potential in the CASTEP module of MS. The binding energy is defined as the following equation:

2 $$E_{\mathbf{b}}\left( \rm{{Li}} \right) = (E_{\rm{{COF}}} + n \times E_{\rm{{Li}}}-E_{{\mathrm{\operatorname{Li-COF}}}}){\mathrm{/}}n$$

where *n* is the number of lithium atoms attached to the pristine COF, and* E*_Li_, *E*_COF_, and *E*_Li‑COF_ are the corresponding energies of the isolated lithium atom, the pristine COF, and Li-inserted COF, respectively. The Gibbs free energy (Δ*G*) was applied to explore and determine the most stable Li-inserted state, which is defined as the following equation:

3 $${\mathrm{\Delta }}G = E_{{\mathrm{\operatorname{Li - COF}}}}-E_{{\mathrm{COF}}}.$$

### MD simulation

The constructed model was optimized by the Forcite module in MS to obtain the initial structure with minimized energy, on the basis of which MD simulations were performed through the Discover module in MS. The corresponding simulation step is 10,000 and the temperature is 298 K.

### Data availability

The data that support the findings of this study are available from the corresponding author on reasonable request.

## Electronic supplementary material

Supplementary Information
