# Supplementary material for: Boosting lithium storage in covalent organic framework via activation of 14-electron redox chemistry
Source: Nat Commun. 2018 Feb 8;9:576. doi: 10.1038/s41467-018-02889-7 (PMC5805684; doi:10.1038/s41467-018-02889-7)
Supplement: Supplementary file 1 — Supplementary Information [file 41467_2018_2889_MOESM1_ESM.pdf]

Supporting Information

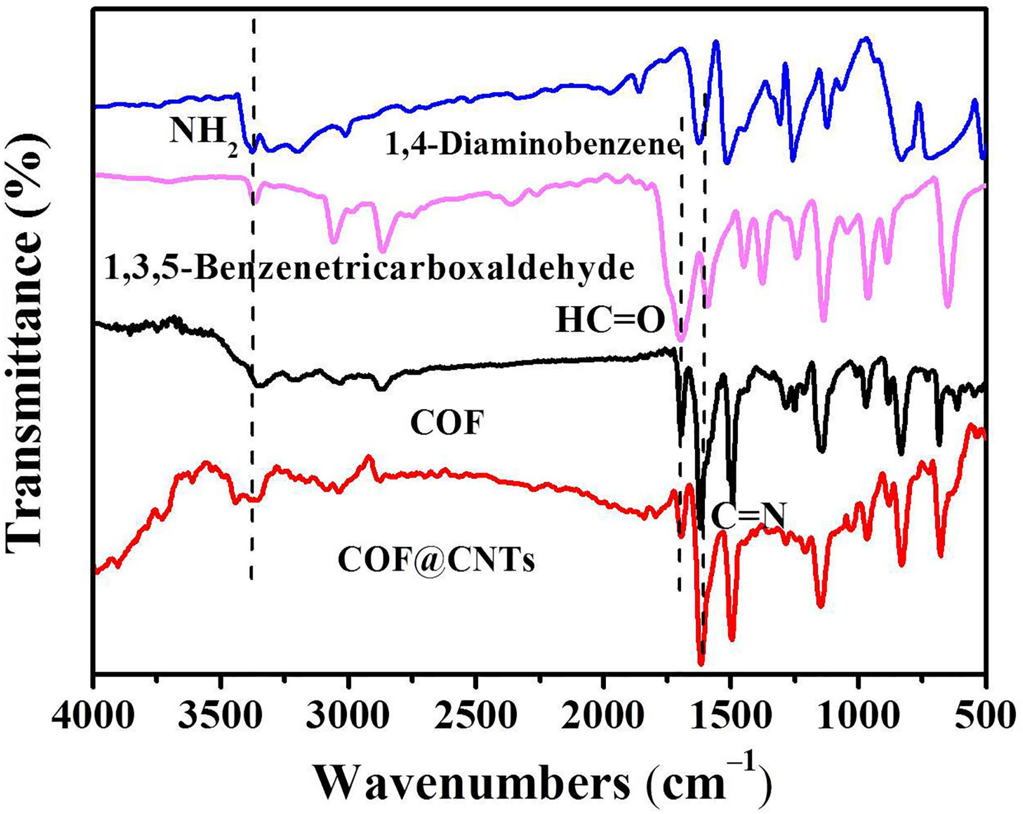

Supplementary Figure 1 | FTIR spectra of 1,4–diaminobenzene, 1,3,5–Benzenetricarboxaldehyde, COF and COF@CNTs. The strong C=N stretch peak can be observed at 1618 cm^{-1} in the FTIR spectrum of COF, indicating the formation of imine bonds from the reaction between the 1,3,5–Benzenetricarboxaldehyde and 1,4–diaminobenzene. Besides, tiny peaks around 1695 and 3415 cm^{-1}, which can be assigned to the terminal aldehyde and amino groups at the edges of the synthesized COFs, can also be observed in the FTIR spectrum. The FTIR spectrum of COF@CNTs is in accordance with that of COF, indicating that the structure integrity of COF in the COF@CNTs. The peaks ranging from 4000 to 3500 cm^{-1} for the COF@CNTs can be assigned to the OH groups from unbound/free hydroxyl or the intermolecular hydrogen bonds between adsorbed water/surface carboxylic and phenolic groups of CNTs, further confirming the successful construction of COF and CNTs in the COF@CNTs composite.

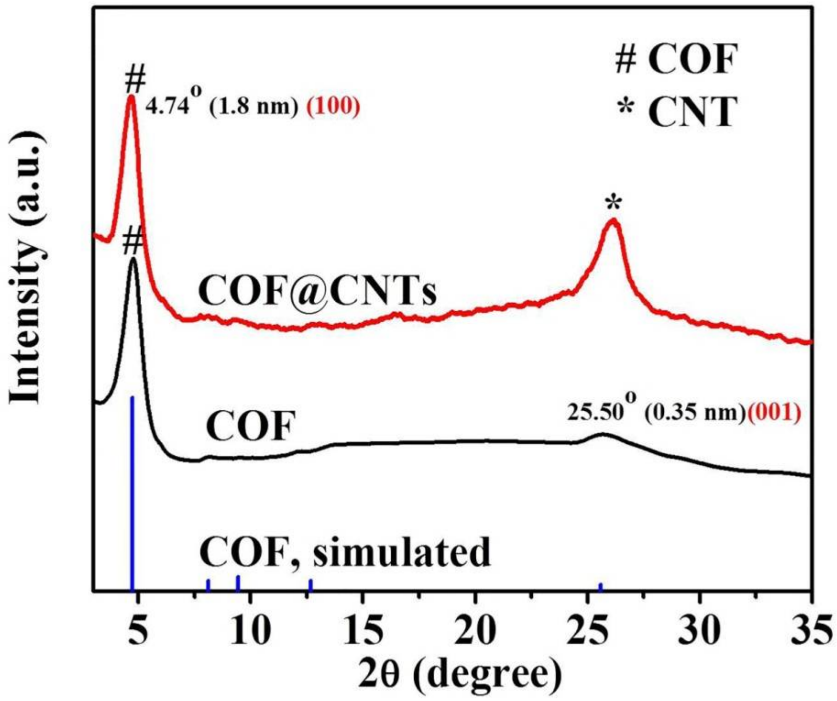

Supplementary Figure 2 | XRD patterns of the as-synthesized COF and COF@CNTs, as well as the simulated XRD pattern of COF. The strong diffraction peaks at 4.74° and 25.50°in the XRD patterns can be assigned to the (100) and (001) planes of COF, which is in accordance with the simulated results. Besides the characteristic peaks for the COFs, a strong peak around 26.1° can also be observed in the XRD pattern of COF@CNTs, which can be assigned to the (002) plane of CNT.

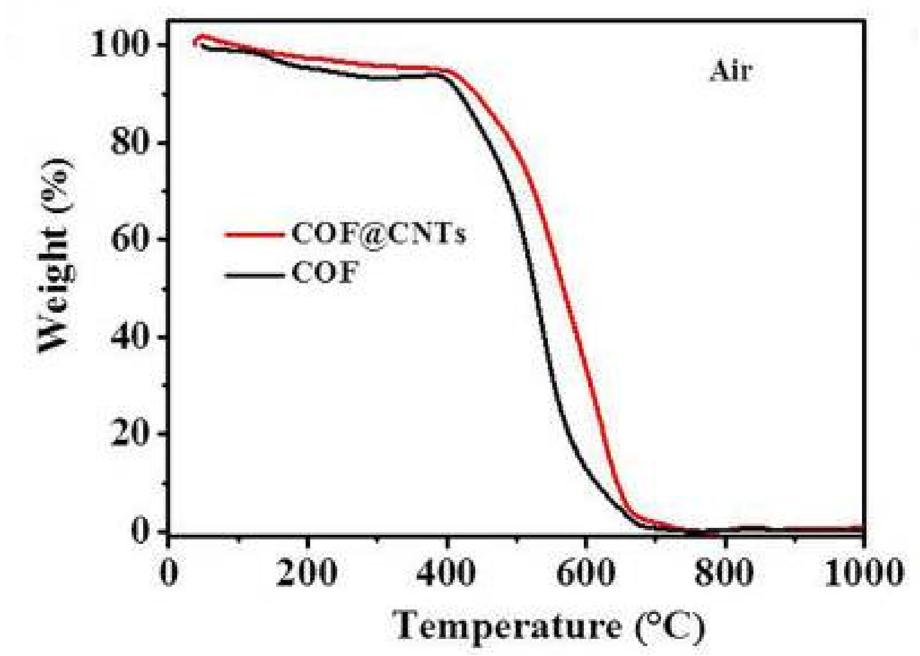

Supplementary Figure 3 | TGA curves of COF and COF@CNTs in air. The weight loss at 200 °C for the COF can be attributed to the removal of absorbed solvent molecules in pore, and the remarkable weight loss between 400 and 700 °C for the COF can be ascribed to the decomposition of COF skeleton. A similar thermal event is also observed for COF@CNTs.

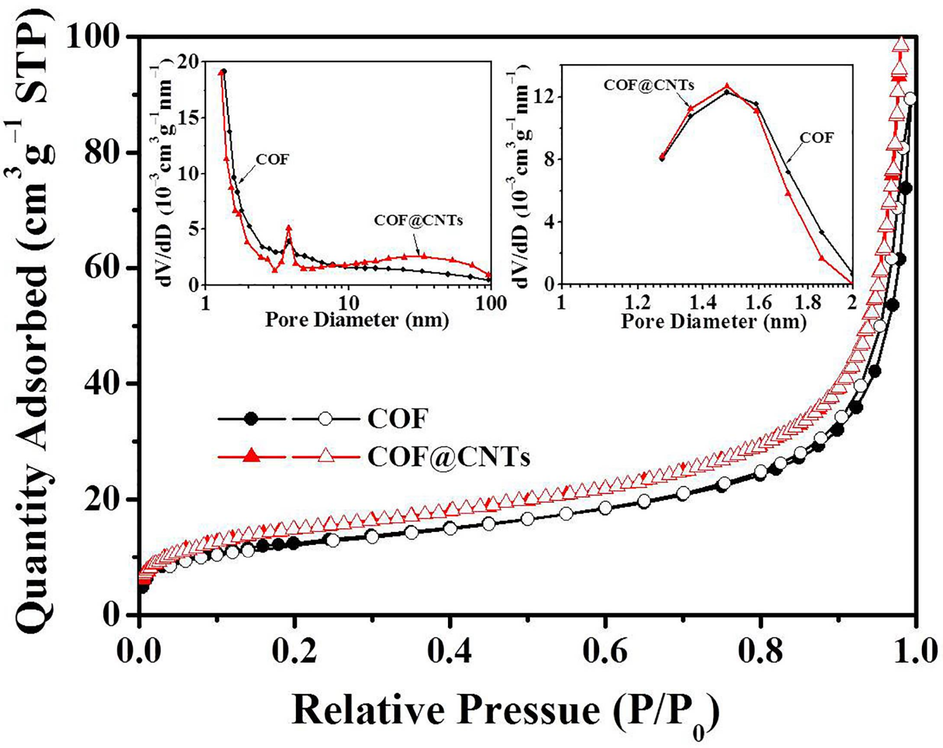

Supplementary Figure 4 | Nitrogen adsorption desorption isotherms of COF and COF@CNTs. The inset is the pore size distribution of COF and COF@CNTs. A BET surface area of 44.36 m^2 g^{-1} and the pore size distribution at ~1.4–1.6 nm are detected for COF, while a larger BET surface area (52.73 m^2 g^{-1}) with similar pore size distribution are achieved for the COF@CNTs.

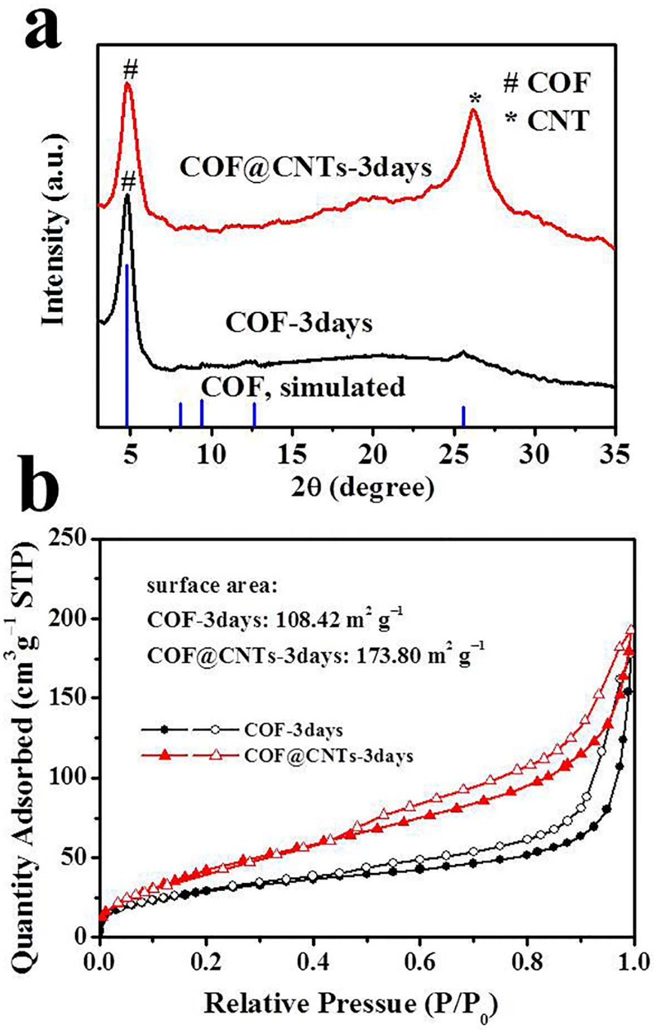

Supplementary Figure 5 | (a) XRD patterns and (b) nitrogen adsorption/desorption isotherms with the specific surface areas of the products of COF-3days and COF@CNTs-3days. The appearance of characteristic peaks for COF (4.7° and 25.5°) and CNTs (26.1°) in the XRD patterns confirm the composition of COF-3days and COF@CNTs-3days. The nitrogen adsorption and desorption measurement indicates BET surface areas of 108.42 m^2 g^{-1} for the COF-3days and 173.80 m^2 g^{-1} for the COF@CNTs-3days, which are substantially larger than the main products of COF and COF@CNTs.

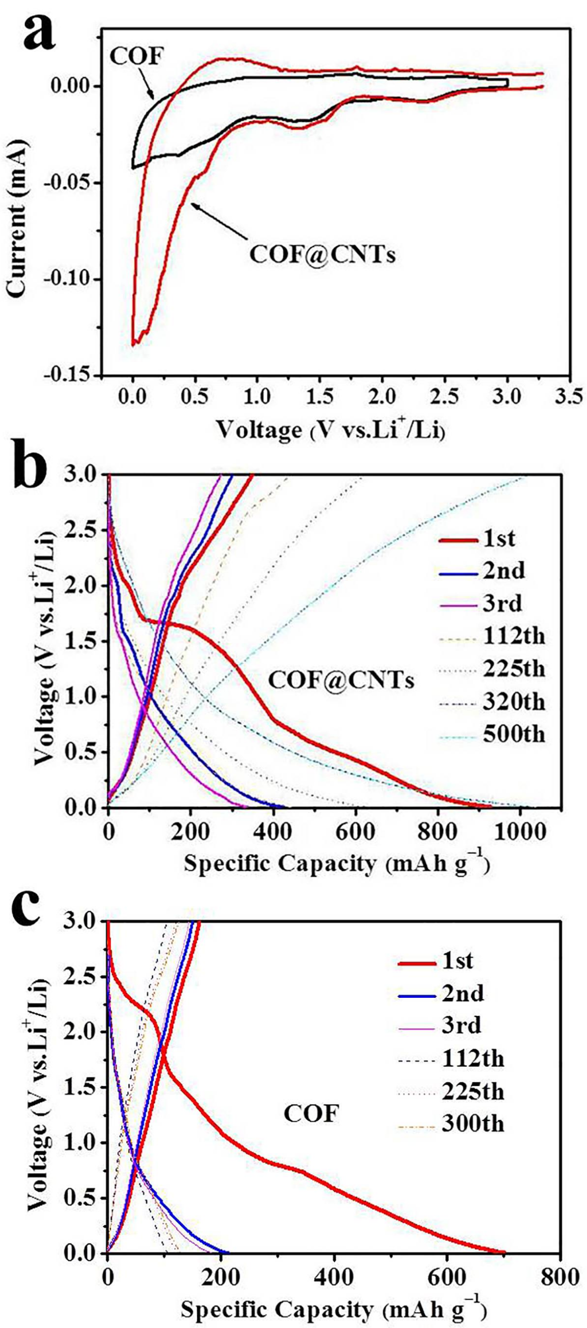

Supplementary Figure 6 | Electrochemical performance of the COF@CNTs: (a) the first-cycle CV curves of the COF@CNTs and COF anodes measured at 0.1 mV s^{-1} between 3.25 V and 0.5 mV. A detected peak at ~1.5 V in the first cathodic scan can be both observed with the COF and the COF@CNTs anodes. This peak is mainly ascribed to the lithium reaction with the C=N units. (b) The discharge (lithium insertion) and charge (lithium extraction) curves of the COF@CNTs at 100 mA g^{-1} for the first three cycles and the 112th, 225th, 320th and 500th cycles. The discharge/charge capacities of the COF@CNTs anode are detected to be 928/383, 446/443, 634/625, 1031/1020 and 1032/1021 mAh g^{-1} for the 1st, 112nd, 225th, 320th and 500th cycles, respectively. (c) The discharge (lithium insertion) and charge (lithium extraction) curves of the bulk COF at 100 mA g^{-1}. The discharge/charge capacities are detected to be 702/163, 214/150, 185/144, 107/105,123/121,129/126 mAh g^{-1} for the 1st, 2nd, 3rd, 112th, 225th, and 300th cycles, respectively. Except for the first three cycles, there is no substantial change for these curves during cycling. Compared to the COF@CNT electrode, a substantially lower reversible capacity observed for the bulk COF electrode should be mainly ascribed to its poor lithium diffusion kinetics.

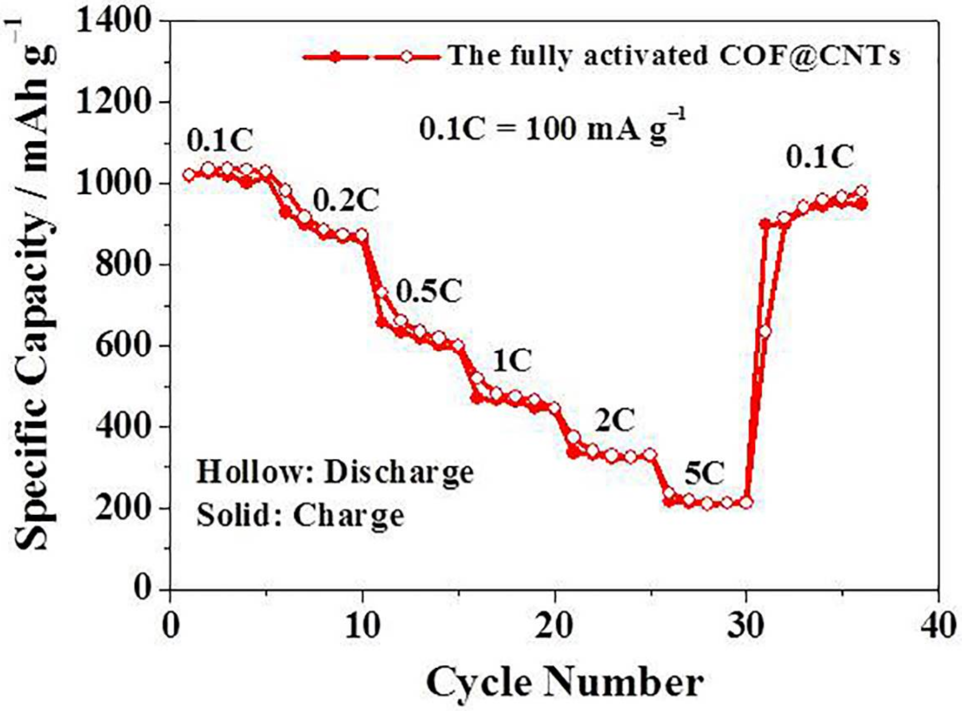

Supplementary Figure 7 | Rate performances of the fully-activated COF@CNTs anode. The average reversible capacities of 1020, 874, 633, 470, 336 and 217 mA h g^{-1} can be detected at current rates of 0.1, 0.2, 0.5, 1, 2 and 5 C (1 C = 1 A g^{-1}), respectively, and a high capacity of 952 mA h g^{-1} can be retained when the current rate returns to 0.1 C.

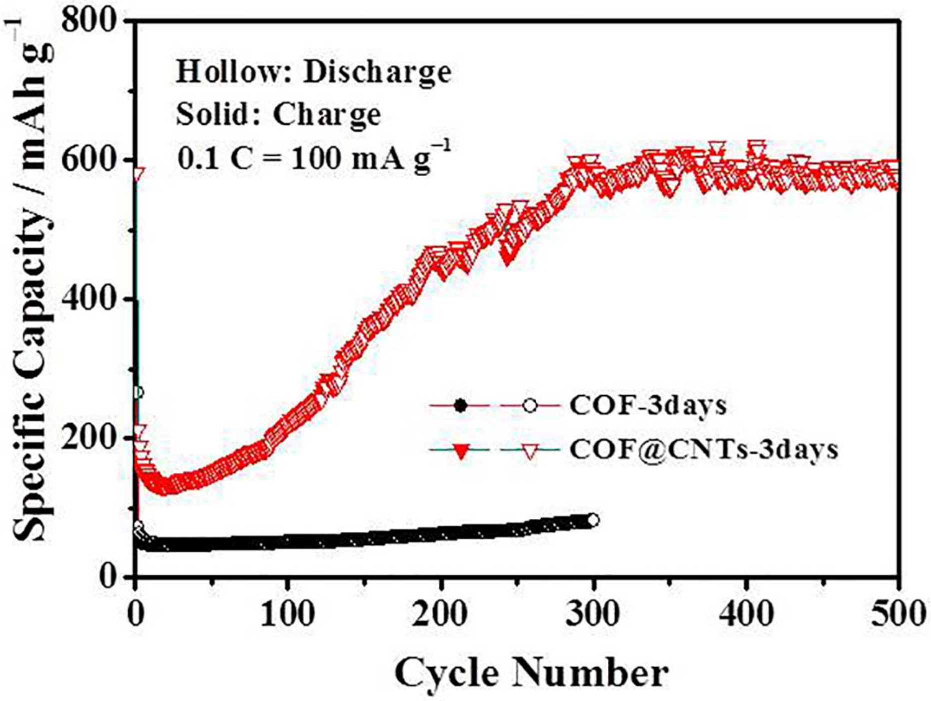

Supplementary Figure 8 | Cycling performances of the anodes of COF-3days and COF@CNTs-3days at 100 mA g^{-1}. For the COF@CNTs-3days anode, initial discharge/charge capacities are detected to be 583/183 mAh g^{-1} with a lower Coulombic efficiency of 31.4 %, and a reversible charge capacity of 571 mAh g^{-1} can be achieved after 500 cycles. A charge capacity of 81 mAh g^{-1} can be detected for the COF-3days anode after 300 cycles. These values are substantially smaller than the main products of COF@CNTs and COF, respectively.

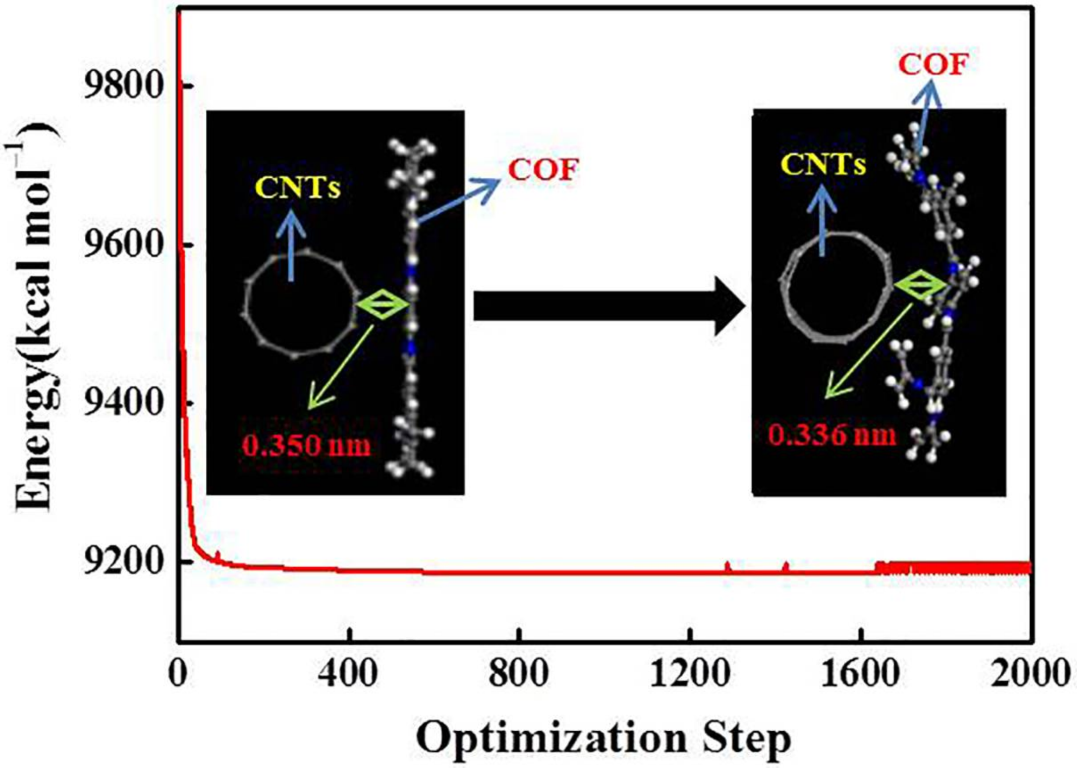

Supplementary Figure 9 | Energy variation during the structural optimization of COF@CNTs. The electrically neutral carbon nanotubes (CNTs) molecular model is built with the dangling bonds at both ends of the CNTs being neutralized by hydrogen atoms. For the COF@CNTs composite, the initial model is established based on the COF molecule placed along the side wall of the CNTs with the distance of 0.350 nm. Through optimizing the model for energy minimization, the COF molecule tends to wrap around the CNTs with the energy decreasing from the initial 9890.045 kcal mol^{-1} to 9185.765 kcal mol^{-1} after 2000 optimization steps.

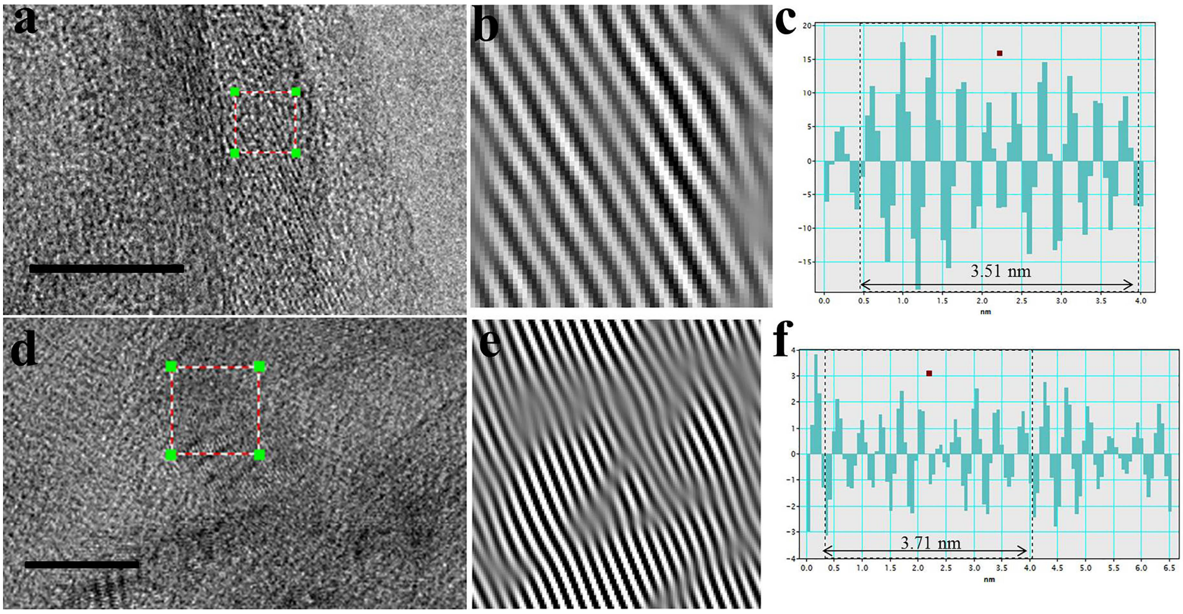

Supplementary Figure 10 | The lattice spacing determination of COF@CNTs after the first cycle: (a) HRTEM image. Scale bar, 10 nm. (b) Lattice fringes of HRTEM image. (c) The lattice spacing (3.51 nm) of ten lattice fringes. The lattice spacing determination of COF@CNTs after 500 cycles: (d) HRTEM image. Scale bar, 10 nm. (e) Lattice fringes of HRTEM image. (f) The lattice spacing (3.71 nm) of ten lattice fringes. In order to calculate the change of *d* value during cycling, ten lattice fringes were measured.

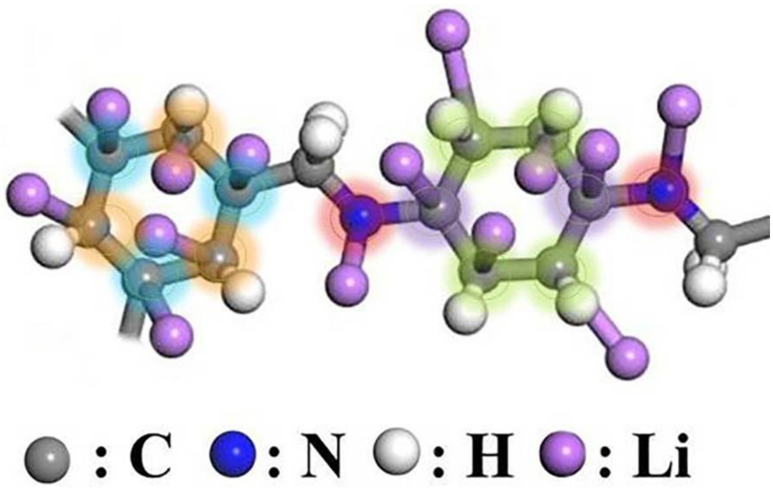

Supplementary Figure 11 | Optimized geometry of COF (gray balls: C; blue balls: N; white balls: H; purple balls: Li) and various possible lithium adsorption sites (nitrogen atom from C=N groups and benzene rings) highlighted with red, yellow, blue, orange and purple colors.

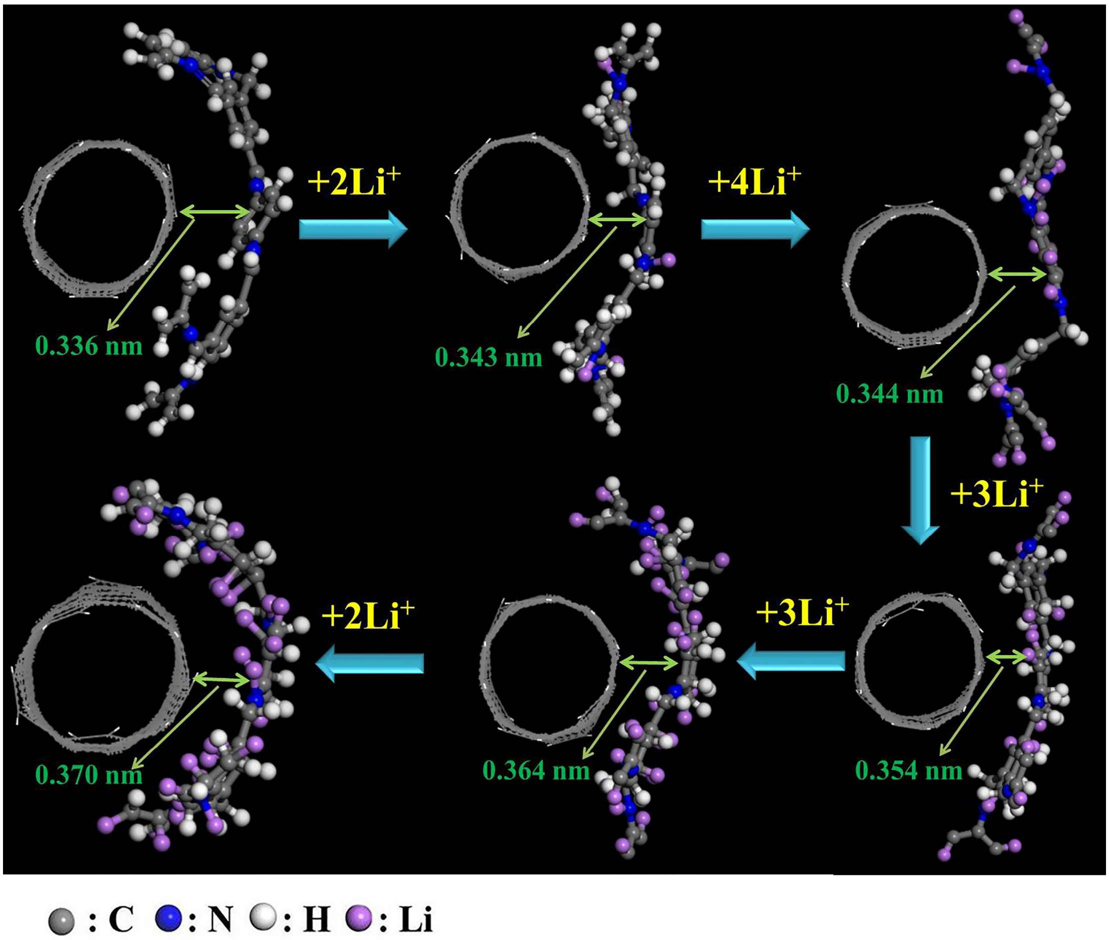

Supplementary Figure 12 | Schematic illustration showing COF wrapping over CNTs during the five-step lithiation process. The distance between COF and CNTs increases slightly during the process of the five-step lithium-ion insertion. Notably, these distances are still in the range of 0.33–0.37 nm, which can be effective distances for π interaction between COF and CNTs.

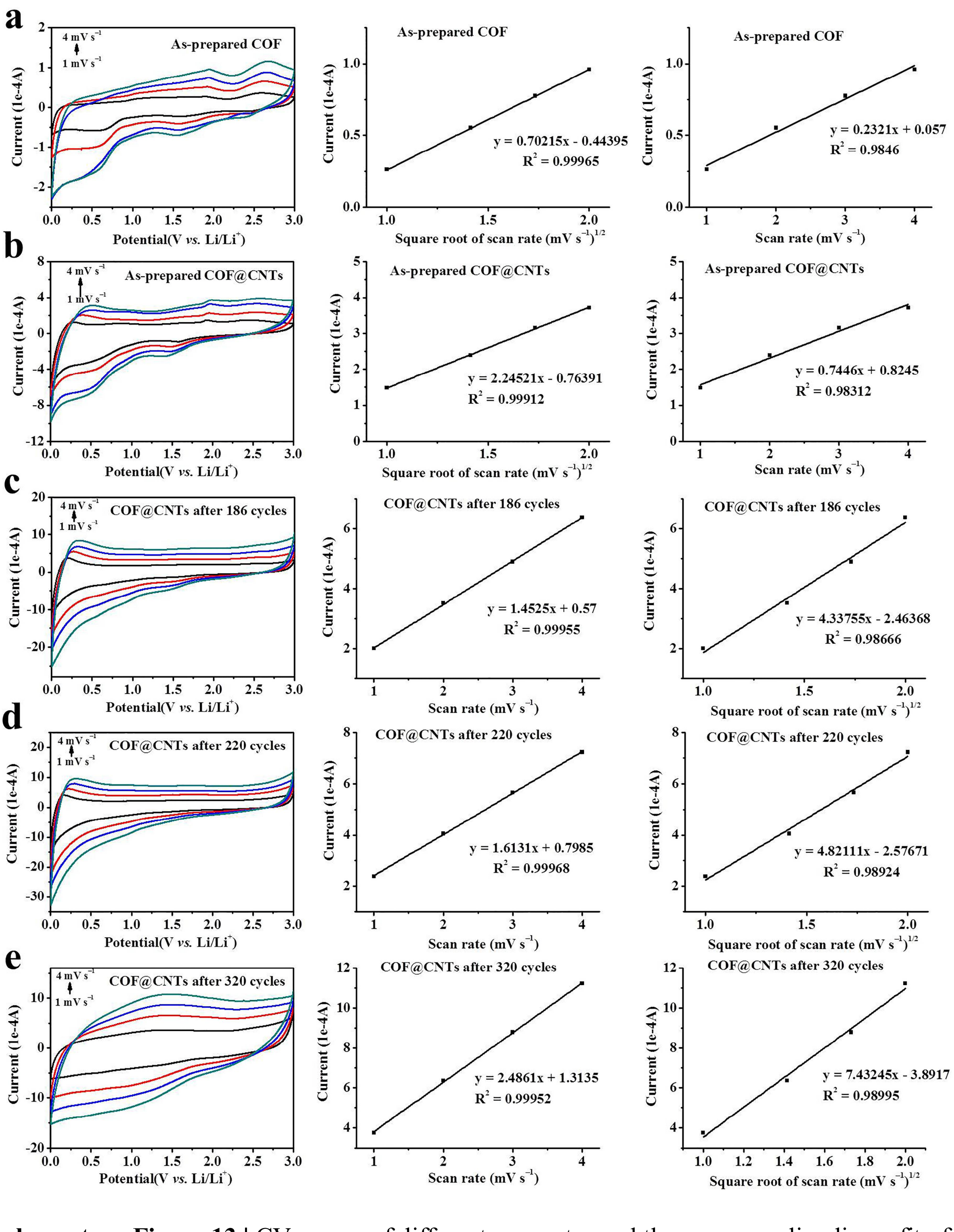

Supplementary Figure 13 | CV curves of different scan rates and the corresponding linear fit of the peak current vs. the scan rate or the square root of scan rate. (a) the as-prepared COF, (b) the as-prepared COF@CNTs, (c) the COF@CNTs after 186 cycles, (d) the COF@CNTs after 220 cycles, (e) the COF@CNTs after 320 cycles.

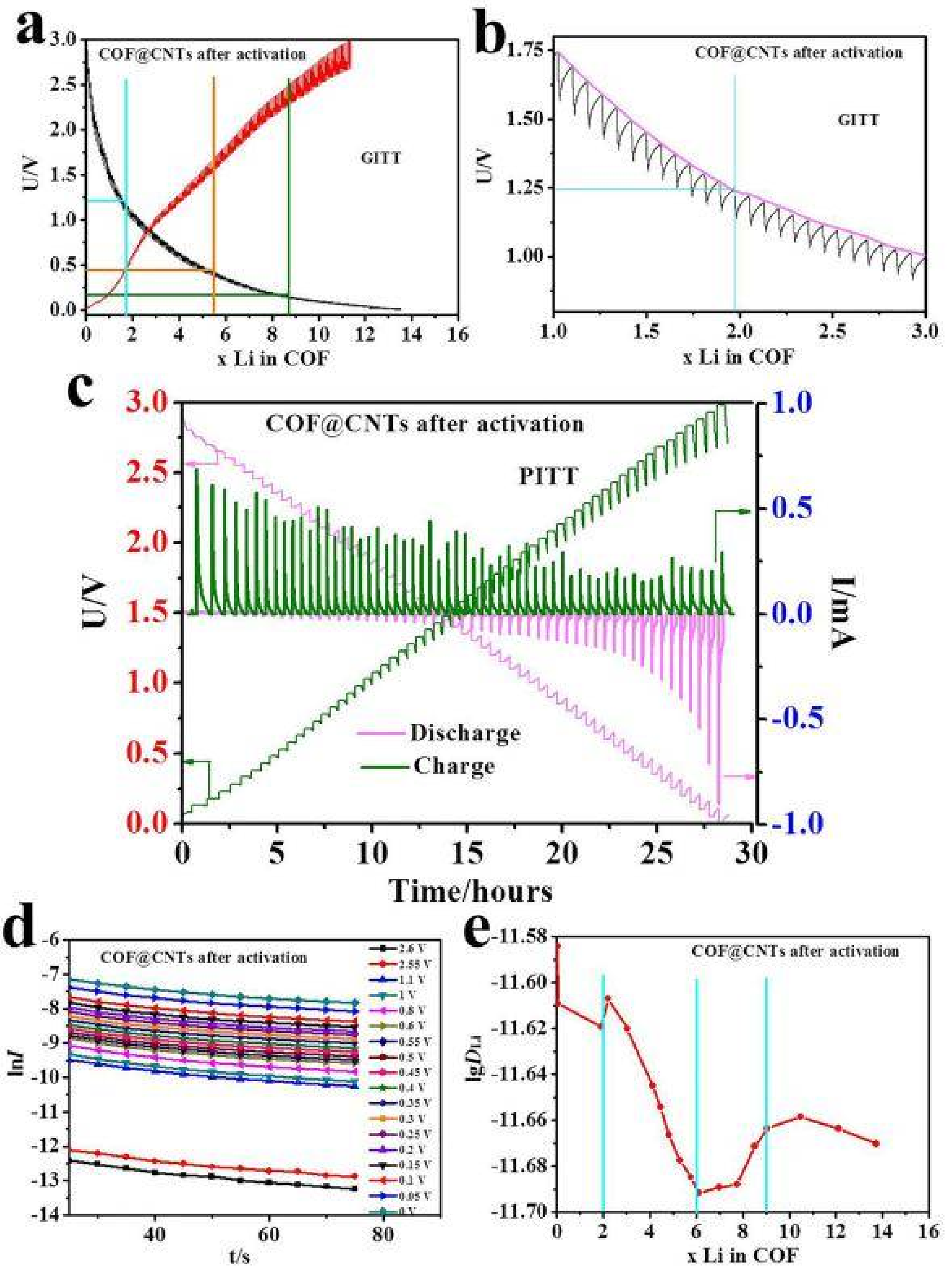

Supplementary Figure 14 | (a) Voltage-composition profile of the fully-activated COF@CNTs electrode discharged to 0 V and recharged to 3.0 V in a GITT mode using an intermittent current rate of C/40 and periodic interruption to reach nearly equilibrium. Several voltage knees can be observed in this figure. (b) The magnified GITT curve corresponds to the two Li^+ storage in COF@CNTs, which corresponds to the lithium storage on two C=N groups. (c) Chronoamperometric responses to stepwise potential scanning (PITT measurements) for the fully-activated COF@CNTs electrode using –50 mV or +50 mV potential steps. (d) Plot of Logarithm of the current vs. time for the COF@CNTs electrode. (e) Plot of chemical lithium diffusion coefficients obtained by PITT as a function of Li-storage content per COF monomer. There is slightly decrease of D_{Li+} when x is increased from 0 to 2 (x corresponds to the number of Li-ion stored in COF). When x=2, the stage is corresponded to the two Li-ion storage on two C=N groups of the COF monomer. After that, the D_{Li+} is increased, which is probably ascribed to the new Li-storage stage on benzene rings. The subsequent decrease/increase of chemical lithium diffusion coefficients D_{Li+} with turning points of x=~6 and x=~9 possibly account for the stepwise Li-storage on benzene rings of the COF monomer.

Supplementary Table 1 Elemental analysis results of pristine COF, CNTs and COF@CNTs

Samples	N (%)	C (%)	H (%)
COF	13.28	73.65	4.53
CNTs	0	94.75	3.65
COF@CNTs	7.75	82.35	2.91

Supplementary Table 2 Discharge capacities of COF@CNTs at different potential stages of different cycles (voltage window of 5 mV to 3 V with an interval of 0.1 V, C: discharge capacity, mAh g^{-1}, V: voltage, V)

COF@CNTs												CNTs	
10th cycle		112th cycle		225th cycle		260th cycle		320th cycle		500th cycle			
C	V	C	V	C	V	C	V	C	V	C	V	C	V
0	3.0	0	3.0	0	3.0	0	3.0	0	3.0	0	3.0	0	3.0
0	2.9	0	2.9	0	2.9	0	2.9	1	2.9	1	2.9	0	2.9
0	2.8	1	2.8	1	2.8	1	2.8	2	2.8	2	2.8	0	2.8
1	2.7	1	2.7	1	2.7	2	2.7	5	2.7	5	2.7	1	2.7
1	2.6	1	2.6	2	2.6	4	2.6	9	2.6	9	2.6	1	2.6
1	2.5	3	2.5	4	2.5	7	2.5	14	2.5	14	2.5	2	2.5
2	2.4	4	2.4	6	2.4	11	2.4	21	2.4	21	2.4	3	2.4
3	2.3	6	2.3	9	2.3	17	2.3	29	2.3	30	2.3	4	2.3
3	2.2	7	2.2	12	2.2	23	2.2	39	2.2	39	2.2	6	2.2
4	2.1	8	2.1	15	2.1	30	2.1	50	2.1	51	2.1	7	2.1
6	2.0	9	2.0	17	2.0	37	2.0	61	2.0	62	2.0	9	2.0
7	1.9	10	1.9	21	1.9	44	1.9	71	1.9	73	1.9	10	1.9
9	1.8	13	1.8	27	1.8	53	1.8	82	1.8	84	1.8	11	1.8
12	1.7	18	1.7	36	1.7	64	1.7	94	1.7	95	1.7	13	1.7
15	1.6	26	1.6	48	1.6	77	1.6	110	1.6	111	1.6	15	1.6
20	1.5	35	1.5	59	1.5	92	1.5	127	1.5	129	1.5	20	1.5
25	1.4	45	1.4	73	1.4	107	1.4	144	1.4	147	1.4	24	1.4
29	1.3	58	1.3	86	1.3	122	1.3	162	1.3	166	1.3	28	1.3
34	1.2	73	1.2	100	1.2	138	1.2	184	1.2	186	1.2	33	1.2
40	1.1	90	1.1	116	1.1	154	1.1	206	1.1	209	1.1	38	1.1
47	1.0	107	1.0	134	1.0	176	1.0	231	1.0	234	1.0	44	1.0
56	0.9	125	0.9	158	0.9	199	0.9	263	0.9	266	0.9	52	0.9
64	0.8	143	0.8	186	0.8	233	0.8	301	0.8	304	0.8	62	0.8
75	0.7	168	0.7	218	0.7	270	0.7	348	0.7	353	0.7	73	0.7
87	0.6	189	0.6	248	0.6	305	0.6	395	0.6	401	0.6	85	0.6
100	0.5	211	0.5	282	0.5	346	0.5	448	0.5	455	0.5	99	0.5
115	0.4	233	0.4	320	0.4	395	0.4	515	0.4	520	0.4	114	0.4
132	0.3	261	0.3	366	0.3	453	0.3	597	0.3	600	0.3	134	0.3
155	0.2	298	0.2	424	0.2	527	0.2	699	0.2	702	0.2	162	0.2
188	0.1	351	0.1	505	0.1	622	0.1	834	0.1	832	0.1	207	0.1
235	0.005	446	0.005	634	0.005	768	0.005	1031	0.005	1032	0.005	303	0.005

Supplementary Table 3 Discharge capacities of COF contribution in the COF@CNTs at different potential stages of different cycles. The discharge capacities of COF contribution at the listed potentials are calculated based on the weighted sum of discharge capacities of COF@CNTs and CNTs in this potential range. (voltage window of 5 mV to 3 V with an interval of 0.1 V, C: discharge capacity, mAh g^{-1}, V: voltage, V)

10th cycle		112th cycle		225th cycle		260th cycle		320th cycle		500th cycle	
C	V	C	V	C	V	C	V	C	V	C	V
0	3.0	0	3.0	0	3.0	0	3.0	0	3.0	0	3.0
0	2.9	0	2.9	0	2.9	0	2.9	2	2.9	2	2.9
0	2.8	1	2.8	1	2.8	1	2.8	3	2.8	3	2.8
0	2.7	1	2.7	1	2.7	3	2.7	8	2.7	8	2.7
0	2.6	1	2.6	2	2.6	6	2.6	14	2.6	14	2.6
1	2.5	3	2.5	4	2.5	11	2.5	22	2.5	22	2.5
1	2.4	5	2.4	8	2.4	17	2.4	34	2.4	34	2.4
1	2.3	6	2.3	11	2.3	26	2.3	47	2.3	48	2.3
1	2.2	7	2.2	16	2.2	35	2.2	63	2.2	63	2.2
2	2.1	8	2.1	20	2.1	46	2.1	81	2.1	82	2.1
3	2.0	9	2.0	24	2.0	57	2.0	98	2.0	100	2.0
5	1.9	10	1.9	29	1.9	68	1.9	115	1.9	118	1.9
8	1.8	14	1.8	38	1.8	83	1.8	133	1.8	136	1.8
11	1.7	22	1.7	52	1.7	101	1.7	152	1.7	154	1.7
15	1.6	34	1.6	71	1.6	122	1.6	177	1.6	179	1.6
20	1.5	47	1.5	88	1.5	144	1.5	204	1.5	207	1.5
25	1.4	60	1.4	107	1.4	166	1.4	229	1.4	235	1.4
30	1.3	79	1.3	128	1.3	190	1.3	258	1.3	264	1.3
36	1.2	101	1.2	149	1.2	213	1.2	292	1.2	295	1.2
41	1.1	127	1.1	172	1.1	237	1.1	325	1.1	331	1.1
49	1.0	151	1.0	199	1.0	270	1.0	364	1.0	370	1.0
58	0.9	176	0.9	233	0.9	305	0.9	413	0.9	419	0.9
66	0.8	201	0.8	275	0.8	355	0.8	472	0.8	478	0.8
76	0.7	235	0.7	321	0.7	410	0.7	544	0.7	552	0.7
89	0.6	264	0.6	365	0.6	462	0.6	616	0.6	627	0.6
102	0.5	291	0.5	413	0.5	523	0.5	698	0.5	709	0.5
116	0.4	317	0.4	467	0.4	595	0.4	801	0.4	809	0.4
130	0.3	352	0.3	531	0.3	681	0.3	927	0.3	932	0.3
150	0.2	394	0.2	611	0.2	787	0.2	1081	0.2	1087	0.2
175	0.1	454	0.1	717	0.1	918	0.1	1281	0.1	1278	0.1
186	0.005	548	0.005	870	0.005	1100	0.005	1551	0.005	1552	0.005

Supplementary Table 4 Discharge capacity contribution of C=N groups and benzene ring of COF during the capacity increase segments in the cycling ranges of the 10–112, 112–225, 225–320 cycles.

Cycle range	Capacity Increment (total)	Capacity Increment (C=N)	Capacity Increment Contribution (C=N)	Capacity Increment (benzene)	Capacity Increment Contribution (benzene)
10–112	362	35	9.7 %	327	90.3 %
112–225	322	47	14.6 %	275	85.4 %
225–320	681	122	17.9 %	559	82.1 %

Supplementary Table 5 The electrochemical property comparison among the COF-relative electrodes for lithium-ion batteries

**Sample**	**D_{TP}–A_{NDI}–COF @CNTs (Ref.25)**	**Tp–DANT–COF (Tb–DANT–COF) (Ref.49)**	**DAAQ–EC OF (Ref.52)**	**N_2–COF (N_3–COF) (Ref.50)**	**TTHPP–COF (Ref.51)**	**COF@CNTs (this work)**
**Pore size**	mesopores (5.06 nm)	micropores (1.6 nm)	mesopores (2.2 nm)	micropores (1.9 and 1.4 nm)	Nanopores (N.A)	micropores (1.8 nm)
**Li-storage group**	C=O	C=O	C=O	C=N	C=O, C–NH, C–S	C=N, aromatic C_6
**Li-storage capacity**	67 mAh g^{-1} after 100 cycles	~72 (80) mAh g^{-1} after 200 cycles	104 mAh g^{-1} after 1800 cycles	~600 (~593) mAh g^{-1} after 500 cycles	~381 mAh g^{-1} after 200 cycles	1536 mAh g^{-1} after 500 cycles
**Test condition**	1.5–3.5 V, 200 mA g^{-1}	1.5–4 V, 200 mA g^{-1}	1.5–4 V, 500 mA g^{-1}	0.05–3 V, 1000 mA g^{-1}	0.005–3 V, 1000 mA g^{-1}	0.005–3 V, 100 mA g^{-1}
**Synthesis method**	Solvothermal synthesis	Solvothermal synthesis	Solvothermal synthesis and ball milling	Solvothermal synthesis	Room-temperat ure synthesis	Room-temperatur e synthesis

Supplementary Note 1

The weight ratio of COF and CNTs in the COF@CNTs was determined to be ~1.4:1, based on the N and C contents in the pristine COF and the COF@CNT composite as shown in Table S1. The two equations can be described as follows:

$$P_{COF}*N_{COF}+P_{CNTs}*N_{CNTs}=N_{COF@CNTs} \quad (1)$$

$$P_{COF}*C_{COF}+P_{CNTs}*C_{CNTs}=C_{COF@CNTs} \quad (2)$$

N_{COF} and C_{COF}, N_{CNTs} and C_{CNTs}, $N_{COF@CNTs}$ and $C_{COF@CNTs}$, correspond to the weight percentages of N and C in pristine COF, pristine CNTs and the COF@CNTs composite, respectively. P_{COF} and P_{CNTs} correspond to the mass percentages of COF and CNTs in the COF@CNTs composite.
